# Exploring Random Forest in Genetic Risk Score Construction

**DOI:** 10.1002/gepi.70022

**Published:** 2025-10-25

**Authors:** Vaishnavi Venkat, Kaylyn Clark, X. Jessie Jeng, Tsung‐Chieh Yao, Hui‐Ju Tsai, Tzu‐Pin Lu, Tzu‐Hung Hsiao, Ching‐Heng Lin, Shannon Holloway, Cathrine Hoyo, Shin‐Yi Chou, Hui Wang, Wan‐Ping Lee, Li‐San Wang, Jung‐Ying Tzeng

**Affiliations:** ^1^ Bioinformatics Research Center North Carolina State University Raleigh North Carolina USA; ^2^ Perelman School of Medicine, Penn Neurodegeneration Genomics Center University of Pennsylvania Philadelphia Pennsylvania USA; ^3^ Department of Pathology and Laboratory Medicine Perelman School of Medicine University of Pennsylvania Philadelphia Pennsylvania USA; ^4^ Department of Statistics North Carolina State University Raleigh North Carolina USA; ^5^ Department of Pediatrics Division of Allergy, Asthma, and Rheumatology, Chang Gung Memorial Hospital Taoyuan Taiwan; ^6^ School of Medicine, Chang Gung University College of Medicine Taoyuan Taiwan; ^7^ Institute of Population Health Sciences, National Health Research Institutes Zhunan Taiwan; ^8^ Department of Medical Science National Tsing‐Hua University Hsinchu Taiwan; ^9^ Institute of Health Data Analytics and Statistics National Taiwan University Taipei Taiwan; ^10^ Department of Public Health National Taiwan University Taipei Taiwan; ^11^ Department of Medical Research Taichung Veterans General Hospital Taichung Taiwan; ^12^ Department of Biological Sciences North Carolina State University Raleigh North Carolina USA; ^13^ Department of Economics Lehigh University Bethlehem Pennsylvania USA

## Abstract

Genetic risk scores (GRS) are crucial tools for estimating an individual's genetic liability to various traits and diseases, computed as a weighted sum of trait‐associated allele counts. Traditionally, GRS models assume additive, linear effects of risk variants. However, complex traits often involve nonadditive interactions, such as epistasis, which are not captured by these conventional methods. In this study, we investigate the use of random forest (RF) models as a model‐free approach for constructing GRS, leveraging RF's capacity to capture complex, nonlinear interactions among genetic variants. Specifically, we introduce two new RF‐based GRS strategies to boost RF performance and to incorporate base data information if available, including (1) ctRF, which optimizes linkage disequilibrium (LD) clumping and *p*‐value thresholds within RF; and (2) wRF, which adjusts the chance of SNP inclusion in tree nodes based on their association strength. Through simulation studies and real data applications of Alzheimer's disease, body mass index, and atopy, we find that ctRF consistently outperforms other RF‐based methods and classical additive models when traits exhibit complex genetic architectures. Additionally, incorporating informative base data into RF‐GRS construction can enhance predictive accuracy. Our findings suggest that RF‐based GRS can effectively capture intricate genetic interactions, and offer a robust alternative to traditional GRS methods, especially for complex traits with nonlinear genetic effects.

## 1 Introduction

Genetic risk score (GRS) provides an estimate of a person's genetic liability to a trait. It is typically computed as a weighted sum of trait‐associated allele counts carried by an individual (Torkamani et al. 2018), where the weights reflect the effect sizes of the alleles. GRS serves as a valuable tool for inferring disease risk, allowing individuals to comprehend their genetic predisposition and translate this insight into actionable steps, such as lifestyle adjustments, preventative interventions, and early detection strategies (Lewis and Vassos 2020). Additionally, GRS aids in understanding the genetic architecture of complex traits, revealing mechanisms of genetic effects, and identifying potential shared signals among different traits (Lewis and Vassos 2020).

Several methods are available for calculating GRS. One widely utilized approach is the “clumping/pruning and thresholding” (C + T) method (Choi and O'Reilly 2019; Euesden et al. 2015; Purcell et al. 2007). To identify the optimal subset of variants to compute GRS, the “C” step in C + T conducts linkage disequilibrium (LD) clumping to eliminate correlated single nucleotide polymorphisms (SNPs) and retains only the most significant trait‐associated SNPs among all correlated variants; the “T” step selects “important” SNPs whose trait‐associated *p* values are less than a certain *p*‐value threshold. The optimal SNP subset for computing GRS is chosen by considering a range of the tuning parameters and selecting the one that maximizes the trait predictive *R* ^2^ in the training data set. Because the C + T GRSs use marginal effect size of a variant as the weights, further improvements have been considered to construct GRS with weights based on the variant effect sizes derived from a joint additive model of all SNPs while accounting for variant LD, for example, lassosum (Mak et al. 2017) and LDPred (Vilhjálmsson et al. 2015). GRS based on joint modeling typically yields higher prediction accuracy owing to the increased accuracy in the estimated SNP effects (Mak et al. 2017).

While these widely adopted GRS approaches have significantly improved risk prediction, they typically assume additive, linear effect mechanisms of risk variants. However, insights from rare variant studies, biological pathways, and genetic networks indicate that both large‐effect rare variants and nonadditive effects, including epistasis, contribute to the genetic architecture of complex traits (Nejentsev et al. 2009; Guindo‐Martínez et al. 2021; Mackay 2014; Mackay and Moore 2014; Singhal et al. 2023; Wei et al. 2014). Intricate gene interactions that influence traits via nonlinear or nonadditive effects have been discovered in various phenotypes, such as insulin resistance (Laakso and Kuusisto 2014; Laakso 2010), schizophrenia (Bergen et al. 2019; Schrode et al. 2019), testosterone levels (Tang et al. 2023), and BMI (Evans et al. 2023). Leveraging methods accounting for complex genetic architectures has the potential to further improve the predictive accuracy of GRS models.

In this study, we investigate the utilities of random forest (RF) (Breiman 2001) for genetic risk prediction. RF is a model‐free, ensemble approach that uses numerous decision trees. It has been shown to have superior performance in genetic risk prediction than other classic machine learning methods, such as penalized regression and support vector machine (SVM) (D. S. W. Ho et al. 2019). Compared to penalized regression, RF does not impose specific model structures, naturally captures complex interactive effects of high‐dimensional risk variants and extends beyond linear and additive effects. Compared to SVM, which relies on “black box” models and numeric input of categorical features, RF stands out for their interpretability as RF provides information on feature importance and each individual tree shows the specific decisions and criteria used to predict outcomes; RF also models SNP genotypes as categorical without the need for preprocessing, such as one‐hot encoding or imposing a specific mode of inheritance, such as additive, recessive, or dominant. Moreover, RF training process benefits from efficient parallelization across multiple CPU cores, a stark contrast to the more demanding SVMs, especially when using kernel methods. The ease of parallel processing with RF is a substantial advantage in the analysis of large‐scale genomic data sets. These advantages have led to extensive RF application in biological research areas, including metabolomics (Kokla et al. 2019), gene expression (Díaz‐Uriarte and Alvarez de Andrés 2006), and genome‐wide association studies (GWAS) (López et al. 2018; Hahn et al. 2022). These findings collectively underscore the effectiveness of RF in delivering robust results from high‐dimensional biological data.

Various attempts have been made to use RF to construct GRS: Botta et al. (2014) develop a T‐Trees algorithm under the RF framework to account for the LD structure among SNPs and enhance predictive performance compared to the standard RF. However, T‐Trees is shown to be outperformed by penalized (logistic) regression with an elastic net penalty in later studies (Privé, Aschard, et al. 2019). Chuang and Kuo (2017) use RF to select candidate SNPs and then build GRS using logistic regression with stepwise selection of the candidate SNPs. Öztornaci et al. (2022) compute GRS by taking the weighted sum of allele counts where the weights are based on the variable importance measurements in RF. While taking advantage of RF's ability to identify important SNPs of various effect mechanisms, the GRS of these recent works still relies on a linear additive aggregate of risk SNPs. Recent work by Lau et al. (2022) demonstrates the direct use of RF for constructing GRS and shows that RF‐based GRS can outperform elastic‐net regression. Their method incorporates LD pruning and uses the probabilistic RF framework proposed by Malley et al. (2012) to estimate disease probabilities within their target cohort (SALIA cohort). However, their study focused on a limited set of SNPS and did not incorporate external base data.

In the common practice of GRS analysis, one can take advantage of trait‐specific information from published base data, which provides reliable GWAS summary statistics estimated from large samples. To build the optimal GRS model to predict the genetic risk of target samples, summary statistics from base data, such as the effect size estimates, are incorporated into target training data to identify the best model parameters and hyperparameters. While base data have benefited contemporary GRS approaches, to the best of our knowledge, none of the RF‐based GRS methods have fully leveraged the extensive summary statistics from base data.

In our study, we introduced two new RF‐based strategies—clumping and thresholding RF (ctRF) and weighted RF (wRF)—to address these limitations. We aim to (1) boost RF performance on polygenic prediction; and (2) incorporate GWAS summary statistics from published base data into RF‐GRS. We conduct various analyses based on simulations and real data. We show that incorporating C + T into RF can greatly enhance prediction accuracy and gain robustness across different levels of etiological homogeneity between base data and target data. To corroborate the simulation findings, we also apply these models to several real data GRS applications, including Alzheimer's disease (AD) analysis using the Alzheimer's Disease Sequencing Project (ADSP) data; body mass index (BMI) analysis using the Taiwan Biobank (TWB) (Fan et al. 2008); and atopy analysis using the Longitudinal Investigation of Global Health in Taiwanese Schoolchildren (LIGHTS) cohort (C. H. Ho et al. 2022; Lu et al. 2020).

## 2 Material and Methods

### 2.1 Random Forest (RF) GRS

We illustrate the RF GRS using binary outcomes, but the framework can be applied to quantitative outcomes straightforwardly. For individual i, the input data include $y_{i}\in R^{1}$, which is the binary outcome such as $y_{i}=1\;$for disease and $y_{i}=0$ otherwise; $X_{i}={\left[x_{i1},\cdots ,x_{\mathrm{iM}}\right]}^{T}\in R^{M}$, which is the M SNP predictors with $x_{\mathrm{im}}=0,1\;$, or 2 being the minor allele count at SNP m, and $Z_{i}\in R^{p}$, which is the p covariates such as population substructures. We use RF to construct GRS by leveraging its ensemble learning capabilities. RF treats the three SNP values of $x_{i}$ as categorical, constructs a total of “ntree” decision trees during training, and outputs the mode of the classes for classification. When growing a tree, it introduces randomness by selecting a subset of samples (via bootstrap) and considering a subset of features in each split (via mtry) so to improve model accuracy and reduce overfitting. Under the RF framework, we set the GRS of an individual as the probability of having disease given SNPs and covariates and estimate the disease probability using the method of Malley et al. (2012) as implemented in the R package “*ranger*” (Wright and Ziegler 2017). There are three major steps involved in estimating the disease probability of an individual: (1) For each terminal node of a tree constructed using non‐OOB samples of the target training data, compute the “disease” proportion in the terminal node by taking the number of disease samples divided by the total number of training samples in that node. (2) For a new individual (from the target testing set) with a given tree, the individual traverses the branches of the tree based on their features until reaching a terminal node. At this terminal node, record the corresponding “disease” proportion of this terminal node computed in Step (1). This step is repeated for all trees in the forest. (3) Estimate the disease probability of this individual by averaging the “disease” proportions across all trees in the forest. For the purpose of GRS, doing so allows each tree to probabilistically classify an individual as “disease” (with probability equal to the “disease” proportion of the final node that the individual falls into) instead of deterministically. The disease probability of an individual estimated in this fashion can avoid too extreme probability estimates when the true disease risk is high (or low), under which every tree tends to deterministically classify an individual as disease (or not disease) and the resulting average would be 1 (or 0).

### 2.2 Incorporating Base Data Information Into RF GRS

To further boost the RF predictive performance and incorporate base data information, we consider two strategies to build RF‐GRS. The first strategy is to integrate C + T (i.e., LD clumping and *p*‐value thresholding) into RF GRS analysis (referred to as *ctRF*). The second strategy is to incorporate SNP association strengths as weights to up‐/down‐weigh the chance of a SNP to be considered as the candidate variables at each split in a tree (referred to as *wRF*). Both strategies require splitting the target data into target 80% training and 20% target testing set; the target training set is used to tune hyperparameters and train the RF‐GRS model.

In ctRF, we first obtain SNP LD information using the training portion of the target data and SNP association *p* values from base data. If base data are unavailable, we compute the *p* values using target training data. To compute these (target training) *p* values, we perform marginal association tests using Wald's test from logistic regression of the binary outcome on each SNP individually, adjusting for covariates if included. Next, we conduct LD clumping and *p*‐value thresholding to select a subset of SNPs to compute RF GRS. The clumping LD *R* ^2^ cutoff and *p*‐value threshold are treated as hyperparameters, in addition to the standard RF hyperparameters (i.e., “mtry,” the number of variables considered at each split, and “ntree,” the number of trees in the forest). The optimal values of these hyperparameters are those maximize Nagelkerke *R* ^2^ (NR2 in short) (Nagelkerke 1991) in the logistic regression model that regresses the outcome $y_{i}$ on the RF GRS using the target training data. To assess the benefits of optimizing both the clumping cutoff and *p*‐value threshold, we also consider two alternative approaches: *cRF*, which only optimizes the clumping cutoff, and *tRF*, which only optimizes the *p*‐value threshold, in addition to the standard RF hyperparameters. To tune the ctRF model, we conduct a grid search over the following hyperparameters: LD clumping *R* ^2^ cutoff, *p*‐value threshold, number of trees (num.trees), number of variables considered at each split (mtry), and minimum node size. For each combination, we compute the RF‐GRS on the target training set, then fit a logistic regression model of the outcome on the RF‐GRS and calculate NR2. The hyperparameter combination yielding the highest NR2 is selected. Although we do not implement an explicit inner cross‐validation loop, RF's out‐of‐bag (OOB) predictions—based on bootstrap‐excluded observations—serve as internal validation and provide a reliable performance estimate for tuning. This approach balances predictive robustness with computational feasibility in high‐dimensional genomic settings.

**Figure 1:**
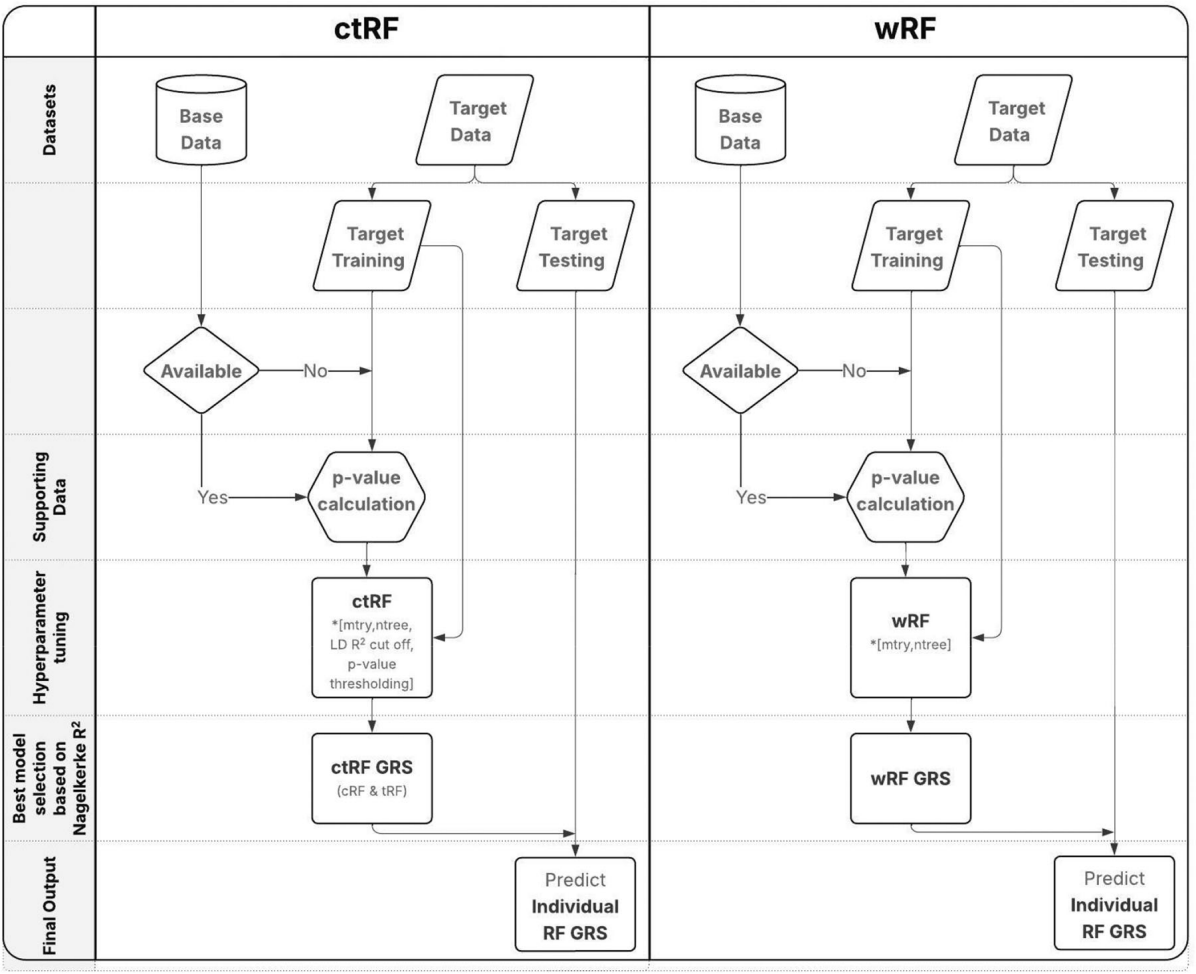
Flowchart of random forest (RF) GRS procedure illustrating the steps for computing RF genetic risk score (GRS). *: [.] indicates hyperparameters.

In wRF, we first obtain the *Z* value for SNP m (denoted as $z_{m})$ based on its association *p* value (denoted as $p_{m})$. The *Z* value is calculated as $z_{m}=|\Phi^{-1}\left(p_{m}/2\;\right)|$, where $\Phi^{-1}\left(\cdot \right)$ is the inverse cumulative density function of the standard normal distribution. Next, we compute the weight of SNP m (denoted as $w_{m}$) using the formula: $w_{m}=\frac{z_{m}-z_{l}}{z_{l}-z_{l}},$ which ensures the weight is between 0 and 1. These weights are then input to “split.select.weights” in the R package “*ranger*,” allowing SNPs with stronger association signals to have a higher probability of being selected among the “mtry” variables at each split in a tree. We illustrate the general RF GRS procedure in Figure 1.

### 2.3 Simulation Design

We conduct simulation studies to evaluate the performance of the proposed RF GRS strategies using real genotype data from Chromosome 21 in the TWB (Fan et al. 2008). Following the preprocessing steps outlined in Supporting S1, there are 10,330 SNPs remaining on Chromosome 21 from 11,654 individuals, where we randomly draw $n_{\mathrm{target}}(=$1000 or 2000) individuals as target samples and treat the remaining $n_{\mathrm{base}}=$10,654 individuals as base samples.

To simulate the binary phenotypes of target samples, we randomly assign 100 SNPs of the 10,330 SNPs as “target causal SNPs,” that is, the causal variants for the target population. This corresponds to roughly 1% of all SNPs and reflects a moderately sparse genetic architecture. Our choice was motivated by standard practice in PRS simulation studies (Choi and O'Reilly 2019; Zhao et al. 2022) and aims to balance biological plausibility with computational feasibility. Let $X_{\mathrm{il}}$ be the minor allele counts of target causal SNP l, l=1,⋯,100, and simulate phenotype ($Y_{i})$ of subject i by considering five different genetic effect scenarios of the causal variants using logistic models. Scenario 1 considers additive main effect of causal SNPs, that is, $\mathrm{logit}\left[P(Y_{i}=1|X_{i})\right]=\;\sum_{l=1}^{100}\beta_{1l}\cdot X_{\mathrm{il}}$ (referred to as Model 1). Scenario 2 considers SNP–SNP interaction effects, that is, $\mathrm{logit}\left[P(Y_{i}=1|X_{i})\right]=\sum_{l=1}^{50}.\sum_{m=51}^{100}{\beta_{2\mathrm{lm}}\cdot X}_{\mathrm{il}}X_{\mathrm{im}}$ where 100 interaction pairs were randomly selected from the full set of 50*50 possible combinations (referred to as Model 2). Scenario 3 assumes a combination of main and interactive effects, that is, $\mathrm{logit}\left[P(Y_{i}=1|X_{i})\right]=\;\sum_{l=1}^{50}\beta_{1l}\cdot X_{\mathrm{il}}+\;\sum_{l=1}^{50}.\sum_{m=51}^{100}{\beta_{2\mathrm{lm}}\cdot X}_{\mathrm{il}}X_{\mathrm{im}}$ where 100 interaction pairs were randomly selected from the full set of 50*50 possible combinations (referred to as Model 3). Scenario 4 considers three‐way SNP interactions among subsets of causal SNPs, that is, $\mathrm{logit}\left[P(Y_{i}=1|X_{i})\right]=\sum_{l=1}^{33}.\sum_{m=34}^{66}.\sum_{m=67}^{99}{\beta_{2\mathrm{lmn}}\cdot X}_{\mathrm{il}}X_{\mathrm{im}}X_{\mathrm{in}}$ where 30 three‐way interaction terms were sampled by combining one SNP from each of three nonoverlapping groups of 33 causal SNPs, reflecting the relative rarity of such higher‐order interactions (referred to as Model 4). Scenario 5 considers quadratic main effects, that is, $\mathrm{logit}\left[P(Y_{i}=1|X_{i})\right]=\;\sum_{l=1}^{100}\beta_{1l}\cdot {X_{\mathrm{il}}}^{2}$(referred to as Model 5). We note that an observable quadratic effect requires that all three genotype groups (0, 1, and 2 copies of the causal allele) are sufficiently represented in the data. When a SNP has a low minor allele frequency (MAF), the rare genotype combinations occur infrequently, making it difficult for the SNP to exhibit a quadratic effect. Therefore, we consider two versions of Scenario 5. Scenario 5a includes all SNPs, the majority of which have MAF < 0.1; Scenario 5b includes only SNPs with MAF > 0.3, ensuring more balanced representation of genotype groups.

We generate effect size β's from Normal (μ,0.1) and then multiply −1 to half of the generated $\beta_{1l}$'s so that ~50% of the causal effect sizes are negative. For main effect size $\beta_{1l}$'s in Model 1 (linear main effects) and Model 5 (quadratic main effects), we set μ=log⁡(1.1) and log(1.5), which corresponds to disease odds ratios of 1.1 and 1.5, respectively; for interaction effect size $\beta_{2\mathrm{lm}}$'s in Model 2 (two‐way interactions) and Model 4 (three‐way interactions), we set μ=log⁡(1.6) and log(2). In Model 3, we consider $\beta_{1l}∼N(\log\left(1.1\right),0.1)$ and $\beta_{2l}∼N(\log\left(1.6\right),0.1)$ as well as $\beta_{1l}∼N(\log\left(1.5\right),0.1)$ and $\beta_{2l}∼N(\log\left(2\right),0.1)$. These μ values are specified on the scale of log odds ratios to capture small and moderate polygenic effects.

We use the same Models 1–5 to simulate the phenotypes of the 10,654 base samples but based on the 100 “base causal SNPs,” which may or may not be the same as the target causal SNPs. Specifically, we allow q causal SNPs to be shared between the base causal SNPs and target causal SNPs. (That is, the base population and target population each contain (100−q) unique causal SNPs while sharing q causal SNPs.) We consider q=100,70,50, and 30, and refer to these scenarios as “BT100,” “BT70,” “BT50,” and “BT30,” respectively. For the q shared causal SNPs, we use the same values of $\beta_{1l}$'s and $\beta_{2\mathrm{lm}}$'s as the target causal SNPs. For the (100−q) unique base causal SNPs, we follow the same procedures to generate effect sizes from normal distribution as done for the target causal SNPs. Once base data is generated, we compute the association *p* values for each of the 10,330 SNPs, and only the association *p* values, rather than the individual‐level base data, are used in the downstream GRS analysis.

Given a set of simulated target individual‐level data and base summary data, we conduct the GRS analysis as follows. First, we partition the target samples into 80% training set and a 20% test set (that is, for $n_{\mathrm{target}}=1000$, this corresponds to $n_{\mathrm{target\_training}}=\;800$ and $n_{\mathrm{target\_testing}}=\;200$). Second, we use the target training samples to derive the GRS model using the proposed RF GRS strategies (i.e., ctRF, tRF, cRF, and wRF). We also apply several other GRS methods as baselines as detailed in the next paragraph. Table S1 summarizes all the GRS methods considered. Finally, we apply the derived GRS models to the target testing set and compute NR2 and area under the ROC curve (AUC) to assess the predictive performance of various methods.

We repeat the above data generation and GRS analysis 100 times. The baseline methods can be classified into two categories. The first category includes five classic, well‐received methods, that is, (1) *oCT*, which is the original Clumping and Thresholding (Choi and O'Reilly 2019; Euesden et al. 2015; Purcell et al. 2007); (2) *pCT*, which is the PCA‐GRS of Coombes et al. (2020) and uses the first principal component (PC) of the candidate GRSs to form the final GRS; (3) *sCT*, which is the Stacked C + T of Privé, Vilhjálmsson, et al. (2019) and uses a penalized linear regression that regresses the trait on all candidate GRSs to identify the optimal linear combination of the candidate GRSs; (4) *LDpred* (Vilhjálmsson et al. 2015); and (5) *lassosum* (Mak et al. 2017). The second category considers penalized linear regression using target data only and does not incorporate base data information, that is, PLR, which is penalized logistic regression with elastic net penalty; and *rfLR*, which uses RF to screen for candidate SNPs and uses stepwise logistic regression to build GRS (Chuang and Kuo 2017). Besides these five baseline GRS methods, we also include original RF (referred to oRF), which applies RF on all SNPs without LD clumping and *p*‐value thresholding, as a benchmark. All baseline methods except pCT are implemented using R package “*bigsnpr*” (Privé et al. 2018). pCT was implemented using a custom script in R while RF was implemented using the R package “*ranger*” (Wright and Ziegler 2017). For all GRS methods, we implement them under the scenario of (1) base data and target data are available (referred to as “BT”) and (2) only target data are available (referred to as “Tonly”).

### 2.4 Real Data Applications

We applied the baseline and proposed methods to compute GRS for three traits: AD, overweight/obesity, and atopy. In the AD GRS analysis, we use ADSP samples as target data, which consist of 11,966 individuals of European and African descent. ADSP is a large‐scale research initiative aimed to identify genetic factors that contribute to AD and related dementias, by sequencing the genomes of a large number of ethnically diverse samples. For base data, we use the study of the International Genomics of Alzheimer's Project (IGAP) (Kunkle et al. 2019), where the GWAS summary statistics for 5,066,495 SNPs were available from https://www.niagads.org/datasets/ng00075 based on 63,926 individuals of European descent (21,982 cases and 41,944 controls). After performing QC (i.e., retaining genotype for a sample with DP > 10 and GQ > 20 and SNPs with a GATK “FILTER” = PASS or in tranche ≥ 99.8%, sample call rate ≥ 80%, supported by < 500 reads, and MAF ≥ 0.01), the target data include 3,587,472 SNPs across 11,966 individuals (6158 cases and 5808 controls); the SNP set is then merged with the base SNP data, resulting in 1,255,048 SNPs. In the AD GRS analysis, we include sex, age, APOE e2, APOE e4, and the first 10 PCs as covariates in GRS model training and evaluation. This analysis allows us to evaluate the performance of the baseline and proposed RF methods when the base summary statistics are obtained from a similar but not identical ethnicity to the target data.

In the overweight/obesity analysis, we consider binary phenotype “overweight/obesity” defined as BMI ≥ 25 kg/m^2^ versus “not” (BMI < 25 kg/m^2^). We use the 11,654 TWB samples as target data (Fan et al. 2008) who have nonmissing BMI and covariate information. TWB is an ongoing large‐scale prospective study of Taiwanese individuals aged 20–70. For base data, we use the summary statistics for BMI from 2,336,269 SNPs in meta‐analyses by the GIANT consortium and UK Biobank (Yengo et al. 2018; Sudlow et al. 2015). These analyses are based on ~700,000 samples of European descent and the data were downloaded from https://portals.broadinstitute.org/collaboration/giant/index.php/GIANT_consortium_data_files. After QC (i.e., removing SNPs with call rates < 0.95, HWE *p* value < 10^−4^, and MAF < 0.05), the target data include 727,208 SNPs across 11,654 subjects (5791 cases and 5863 controls). The SNP set is then merged with the base SNP data, resulting in 506,533 SNPs. In the overweight/obesity GRS analysis, we include sex, age, and the first 10 PCs as covariates in the GRS model training and evaluation. This analysis allows us to evaluate the performance of the baseline and proposed RF methods when the base summary statistics are obtained from a different ethnicity than the target data.

In the atopy GRS analysis, we obtain our target data from LIGHTS data set (C. H. Ho et al. 2022; Lu et al. 2020), which consists of 1292 individuals. LIGHTS is a longitudinal project aimed to study the health conditions and growth development of Taiwanese school‐aged children aged 5–8 years. We conduct target‐only (Tonly) analysis due to the limited availability of large‐scale GWAS summary statistics for atopy in compatible age groups. After QC (i.e., removing SNPs with call rates < 95%, HWE *p* value < 10^−6^, and MAF < 0.01), the data set included 448,924 SNPs across 1292 Taiwanese children (843 atopic cases and 449 controls). In the atopy GRS analysis, we include sex, age, and the first 10 PCs as covariates in GRS model training and evaluations. The atopy analysis allows us to evaluate the performance of the baseline and proposed RF methods when the base summary statistics are unavailable.

For all analyses, the target samples are randomly divided into 80% training and 20% testing subsets. To mitigate potential bias introduced by random data set partitioning, we conduct 5‐fold cross‐validation. We optimize the tuning parameters by selecting the best NR2 value in the target training data set and calculate the prediction NR2 for each target testing set. The average NR2 value across these five folds are then reported.

## 3 Results

### 3.1 Simulation Results

#### 3.1.1 Scenario 1—Additive SNP Main Effects

**Figure 2:**
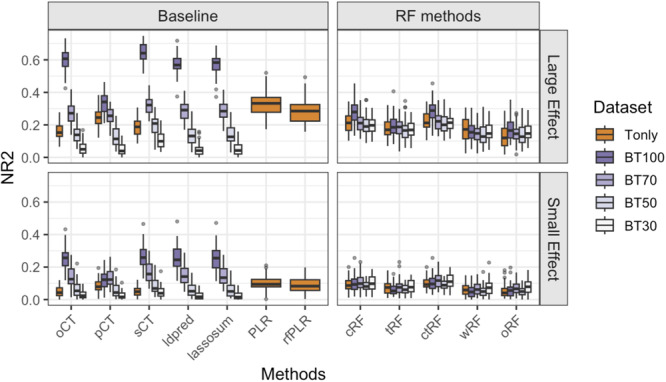
Boxplots of the Nagelkerke *R* ^2^ (NR2) values for different GRS methods, based on 100 simulation replications under Simulation Scenario 1, where causal SNPs exhibit additive main effects on the outcome. “Tonly” indicates analysis using only target data; “BT q” indicates analysis using both base and target data, where q = 100, 70, 50, and 30 denote the number of causal SNPs shared in common between the base causal SNPs and target causal SNPs out of the 100 base causal SNPs. The target sample size $n_{\mathrm{target}}$ = 1000.

The results of Scenario 1 are shown in Figure 2. When only using target data (i.e., Tonly; orange boxplots), we observe that GRS using logistic regressions (i.e., PLR and rfLR) perform the best, which is expected because linear models are the true underlying effect mechanisms. Regardless of the effect sizes, ctRF has lower NR2 than PLR and rfLR but is the next best method, closely followed by sCT, and then pCT and oCT. Among the RF‐based methods, (1) ctRF has a similar NR2 as cRF and slightly higher NR2 than tRF, which shows the importance of optimizing clumping cutoffs and *p*‐value thresholds and that tuning clumping cutoffs may be more beneficial than *p*‐value thresholds. (2) wRF (i.e., up‐weighting/down‐weighting the chance of an SNP to be considered in a tree split based on the GWAS association strengths) does not perform as well as ctRF (or cRF and tRF), although it has slightly higher or similar NR2 compared to oRF.

When incorporating base data that is highly informative (i.e., BT100; dark purple boxplots), linear‐regression‐based methods that account for base information (i.e., lassosum, LDpred, and CT‐based methods) can have substantially higher NR2 than the “golden standard” methods (PLR and rfLR) which do not use base data information. Among the RF‐based methods for BT100, incorporating base information can improve NR2 compared to their Tonly counterparts except for wRF. However, ctRF‐BT100, which is the best‐performing RF‐based method, still only has comparable or lower NR2 compared to PLR and rfLR. When the base data become less informative (e.g., BT70, BT50, and BT30, where the number of shared causal SNPs between the base and target data sets decreases from 100 to 70, 50, and 30, respectively), NR2s drop for all methods compared to BT100. PLR and rfLR, which do not rely on base information, outperform other BT‐based models under BT50 and BT30.

We report AUC in Figure S1. The relative performance of different methods based on AUC is similar to that of NR2 although the AUC variations in cRF, tRF, and ctRF are much larger than wRF and oRF.

#### 3.1.2 Scenario 2—Two‐Way SNP–SNP Interaction Effects

**Figure 3:**
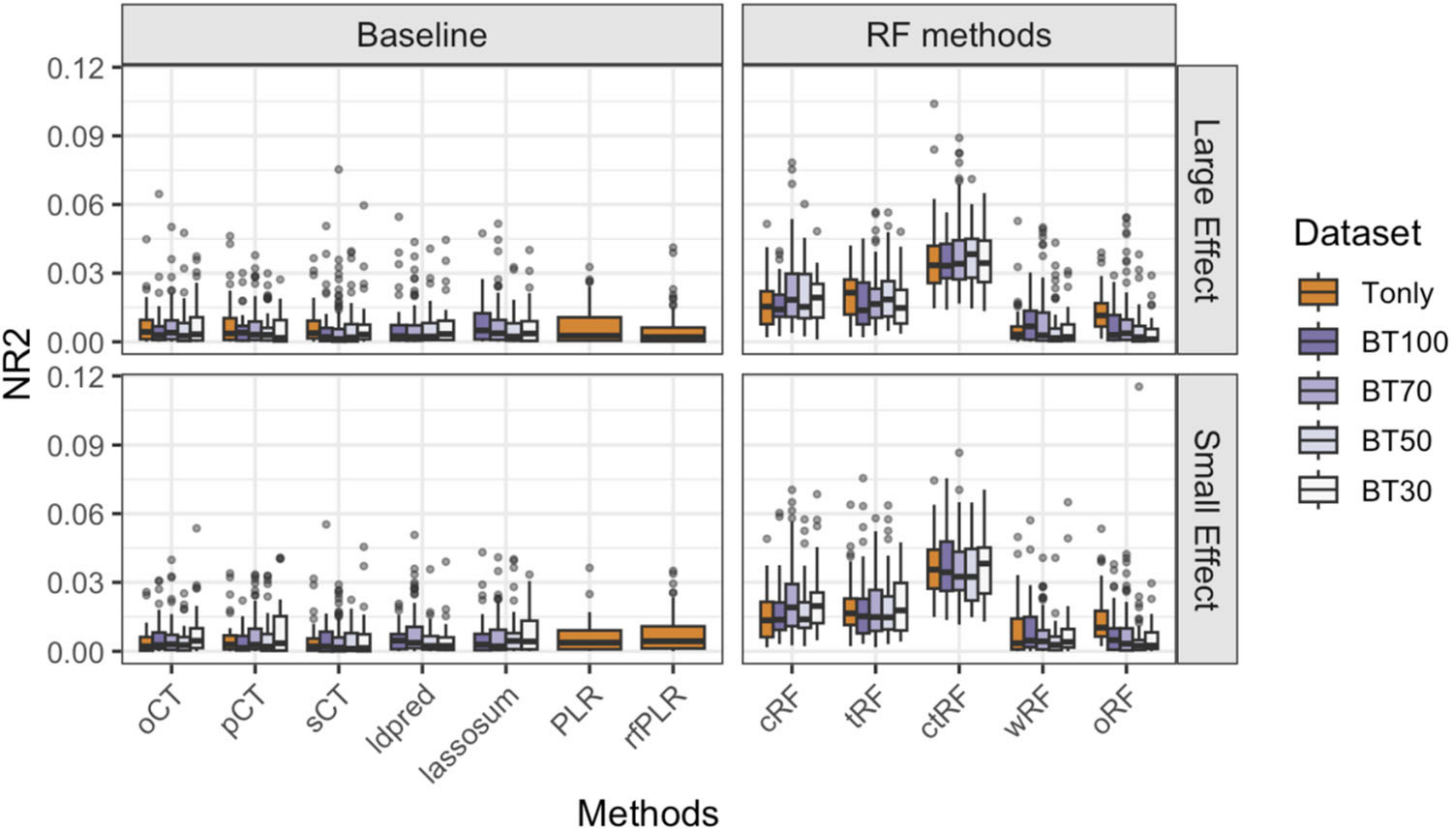
Boxplots of the Nagelkerke *R* ^2^ (NR2) values for different GRS methods, based on 100 simulation replications under Simulation Scenario 2, where causal SNPs exhibit two‐way SNP–SNP interaction effects on the outcome. “Tonly” indicates analysis using only target data; “BT q” indicates analysis using both base and target data, where q = 100, 70, 50, and 30 denote the number of causal SNPs shared in common between the base causal SNPs and target causal SNPs out of the 100 base causal SNPs. The target sample size $n_{\mathrm{target}}$ = 1000.

The results of Scenario 2 are shown in Figure 3. Our key findings under this scenario include: (1) ctRF consistently outperforms all baseline methods, regardless of whether base information is incorporated. (2) Because the base information is derived from marginal association tests, it may not be informative for interactive SNPs, even under the scenario of BT100. Consequently, across all GRS methods, we observe no obvious gain under BT100 compared to Tonly, and the relative performance among BT100, BT70, BT50, and BT30 appears somewhat random. (3) Among the RF‐based methods, the relative performance is similar to what is observed in Scenario 1—ctRF performs the best and has substantially higher NR2 than cRF and tRF, highlighting the importance of identifying optimal LD cutoff and *p*‐value threshold. As in Scenario 1, wRF does not provide much gain compared to the baseline GRS methods under the interactive effect mechanism and can even yield lower NR2 than oRF. The results of AUC are shown in Figure S2, where the relative performance of different methods is similar to that of NR2.

#### 3.1.3 Scenario 3—Mixture of Additive Main Effects and Interactive SNP Effects

**Figure 4:**
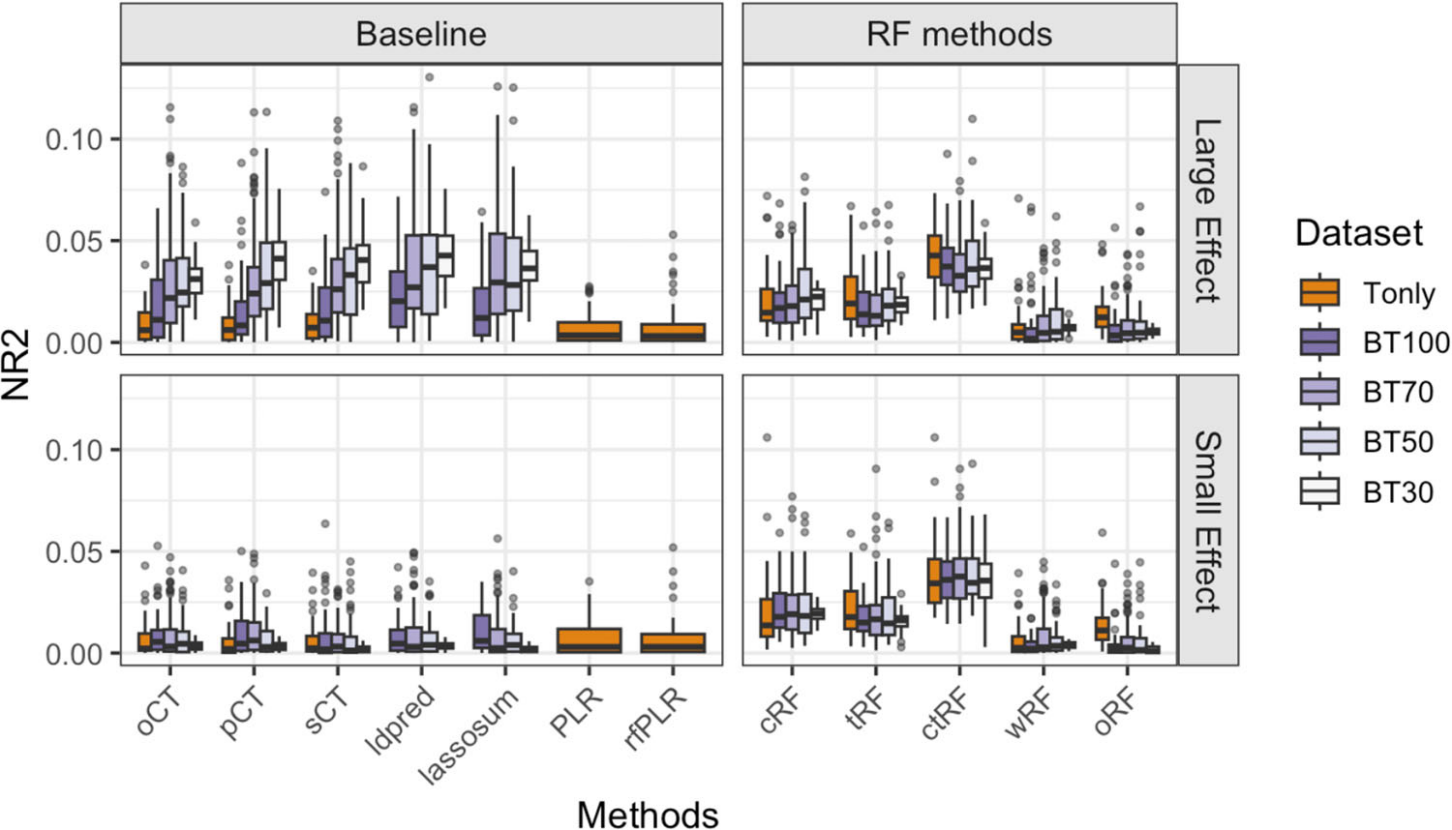
Boxplots of the Nagelkerke *R* ^2^ (NR2) values for different GRS methods, based on 100 simulation replications under Simulation Scenario 3, where causal SNPs exhibit a combination of main and interactive effects on the outcome. “Tonly” indicates analysis using only target data; “BT q” indicates analysis using both base and target data, where q = 100, 70, 50, and 30 denote the number of causal SNPs shared in common between the base causal SNPs and target causal SNPs out of the 100 base causal SNPs. The target sample size $n_{\mathrm{target}}$ = 1000.

The results of Scenario 3 are shown in Figure 4. The results are similar to Scenario 2—(1) ctRF consistently yields the best performance among all RF‐based methods; and (2) RF‐based methods, except for wRF, outperform all baseline models, regardless of the availability of base data information or how many causal SNPs are shared between base and target data. The results of AUC are shown in Figure S3, where the relative performance of different methods is similar to that of NR2, except that wRF and oRF have more comparable AUC to cRF, tRF, and ctRF.

#### 3.1.4 Scenario 4—Three‐Way SNP–SNP Interaction Effects

**Figure 5:**
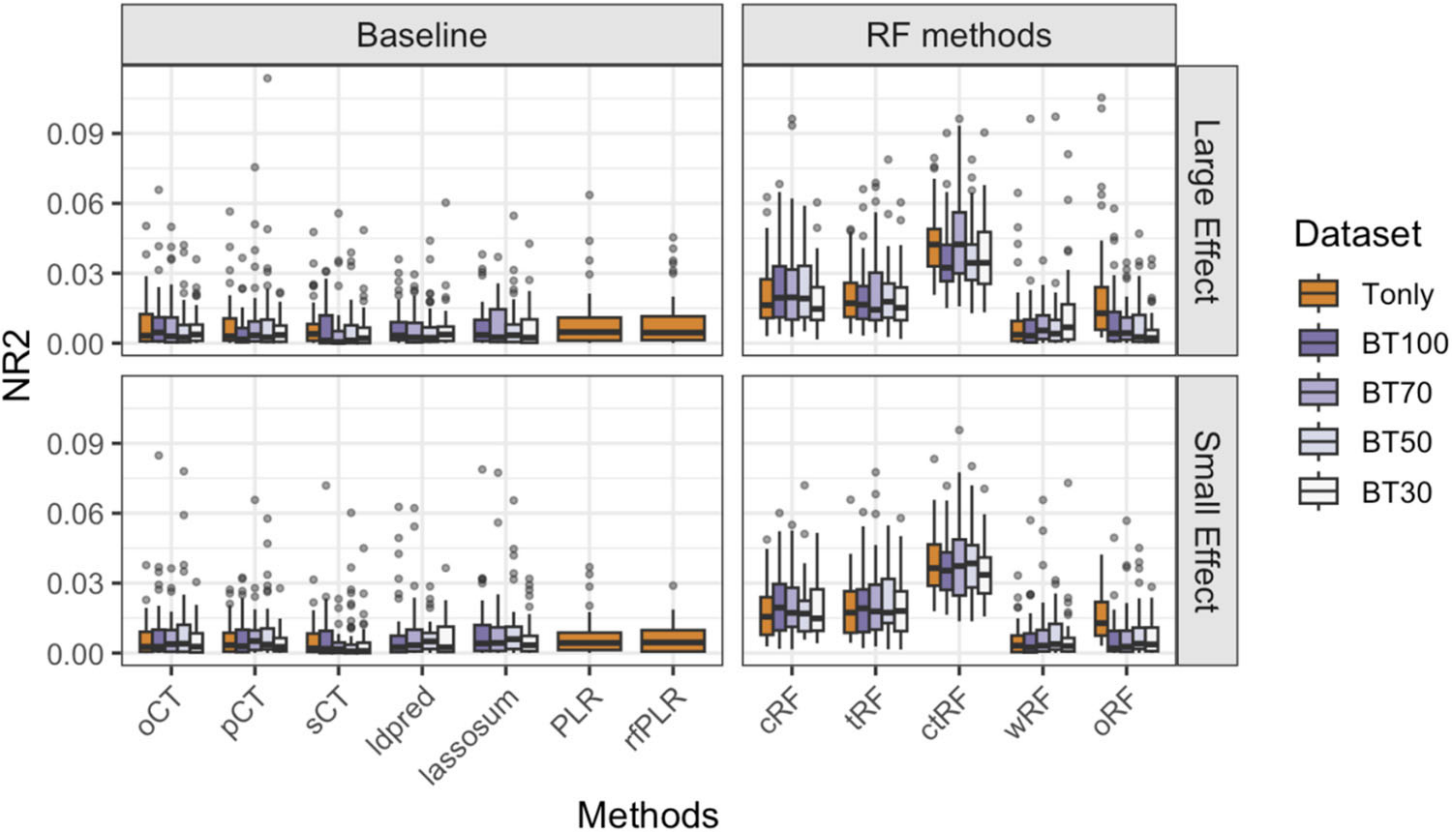
Boxplots of the Nagelkerke *R* ^2^ (NR2) values for different GRS methods, based on 100 simulation replications under Simulation Scenario 4, where causal SNPs exhibit three‐way SNP–SNP interaction effects on the outcomes. “Tonly” indicates analysis using only target data; “BT q” indicates analysis using both base and target data, where q = 100, 70, 50, and 30 denote the number of causal SNPs shared in common between the base causal SNPs and target causal SNPs out of the 100 base causal SNPs. The target sample size $n_{\mathrm{target}}$ = 1000.

The results of Scenario 4 are shown in Figure 5 and are similar to Scenario 2 (two‐way SNP–SNP interactions). That is, we continue to observe that the performance of RF‐based methods is generally superior to baseline methods in the large‐effect panel, with ctRF consistently achieving the highest NR2 among all RF variants across all base–target overlap conditions. As seen in previous scenarios, wRF does not provide a substantial advantage compared to the other RF methods. The AUC results (Figure S4) show similar patterns to NR2, although the gap between wRF/oRF and cRF/tRF becomes smaller.

#### 3.1.5 Scenario 5—Quadratic SNP Main Effects

The results of Scenario 5 are presented in Figures S5 (Scenario 5a—based on all SNPs) and S6 (Scenario 5b—based on SNP with MAF > 0.3). We observe that NR2 and AUC generally show similar relative performance across methods, though oRF and wRF perform relatively better in AUC; moreover, performance remains sensitive to the frequencies of causal alleles. When the causal allele frequencies are low (Figure S5), there are very few individuals with two copies of causal alleles, and hence quadratic effects are not obvious; consequently, GRS methods assuming linear effects (e.g., PLR and rfPLR) perform the best, closely followed by ctRF. On the other hand, when the causal allele frequencies are high (e.g., > 0.3; Figure S6), the quadratic effects become obvious and RF‐based GRS methods perform better than baseline methods, especially ctRF. The results support the conclusion that linear methods are better suited for additive effects, whereas RF‐based methods gain relative advantage in more complex, nonlinear architectures.

All results presented here are based on simulations with a target sample size of $n_{\mathrm{target}}$ = 1000 individuals. To assess robustness to increased sample size, we additionally performed simulations with $n_{\mathrm{target}}$ = 2000 for Scenarios 1–4; results are shown in Figures S7–S10 and are broadly similar to those with 1000 samples, with slightly greater increases in NR2 than in AUC. We also examined the optimized LD clumping and *p*‐value thresholds selected across different scenarios. In general, we observe that higher LD clumping thresholds combined with relatively relaxed p‐value filtering tend to yield better predictive performance than overly stringent filtering, although this is a general trend and not a strict rule. The limited performance of cRF and tRF, which optimize only one of these hyperparameters, further underscores the importance of jointly tuning both clumping and *p*‐value thresholds—as done in ctRF—for robust performance. Additionally, we note that Scenarios 1 and 5a consistently retained the largest number of SNPs after clumping. Similarly, these scenarios tended to favor lower *p*‐value thresholds. In contrast, for other models, we observed that the optimal grid search settings often favored higher *p*‐value thresholds. This likely allows retention of a broader set of SNPs that may participate in capturing interaction effects.

In summary, the simulation results indicate that RF may not be as effective for predicting traits influenced by additive genetic architecture—as observed in Scenario 1, where ctRF does not show a significant advantage over linear‐model baseline methods. However, for traits influenced by more complex genetic mechanisms, such as Scenarios 2–5, ctRF exhibits clear potential to outperform baseline models. This advantage persists regardless of the availability of base data. The results also suggest that RF‐based methods could benefit from careful tuning of hyperparameters, such as LD clumping and *p*‐value thresholds, and that incorporating base information may further improve predictive performance.

### 3.2 Real Data Results

We included oRF in the simulation study as a benchmark but did not extend it to the real data applications for two main reasons. First, in our simulation results (Figures 2, 3, 4, 5), oRF consistently showed lower predictive performance than ctRF, especially when the underlying genetic architecture involved additive or interaction effects. This suggests that simply fitting RF to all SNPs without LD pruning or *p*‐value filtering is not competitive. Second, applying oRF directly to genome‐wide SNP data in real studies is computationally prohibitive and can be unstable in high‐dimensional settings.

**Figure 6:**
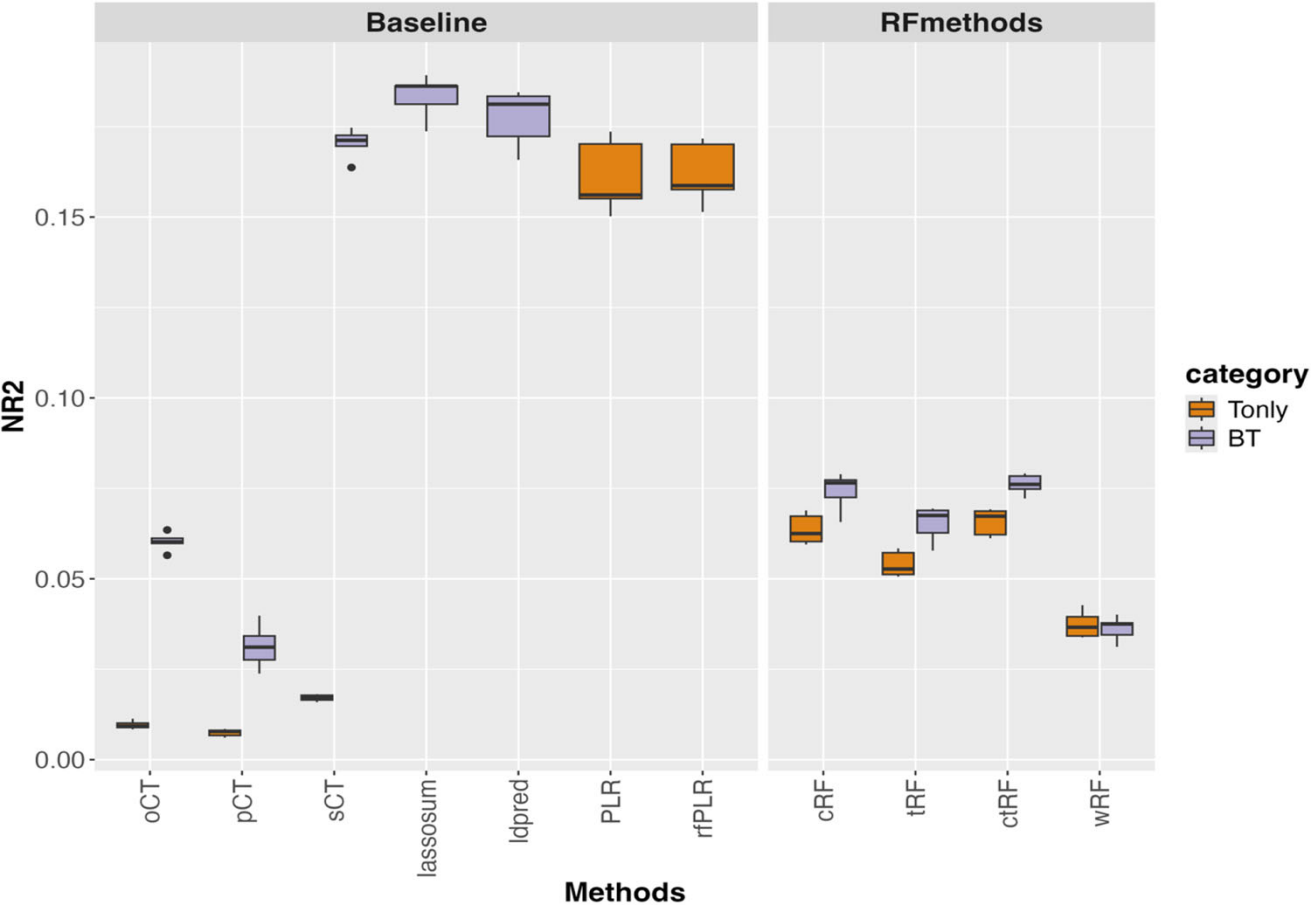
GRS analysis for Alzheimer's disease, using ADSP samples (individuals of European descent and African descent) as target data and IGAP summary statistics (individuals of European descent) as base data. The figure shows boxplots of the Nagelkerke *R* ^2^ (NR2) values for different GRS methods, obtained from 5‐fold cross‐validation.

Figure 6 shows the prediction accuracy of different GRS methods in the AD GRS analysis, using ADSP samples as target data and IGAP samples as base data. In the Tonly analysis, PLR and rfLR are the top‐performing methods, with NR2 values higher than those of RF‐based methods, suggesting a possible additive polygenic effect in AD. Incorporating base information further increases NR2 compared to the Tonly analysis, and GRS methods that incorporate base information and use a joint model framework (i.e., LDpred and lassosum) achieve the highest NR2 among all methods. This implies that the base data obtained from individuals of European descent are still informative for AD‐associated SNPs in the ADSP samples, which include individuals of European descent and African descent.

**Figure 7:**
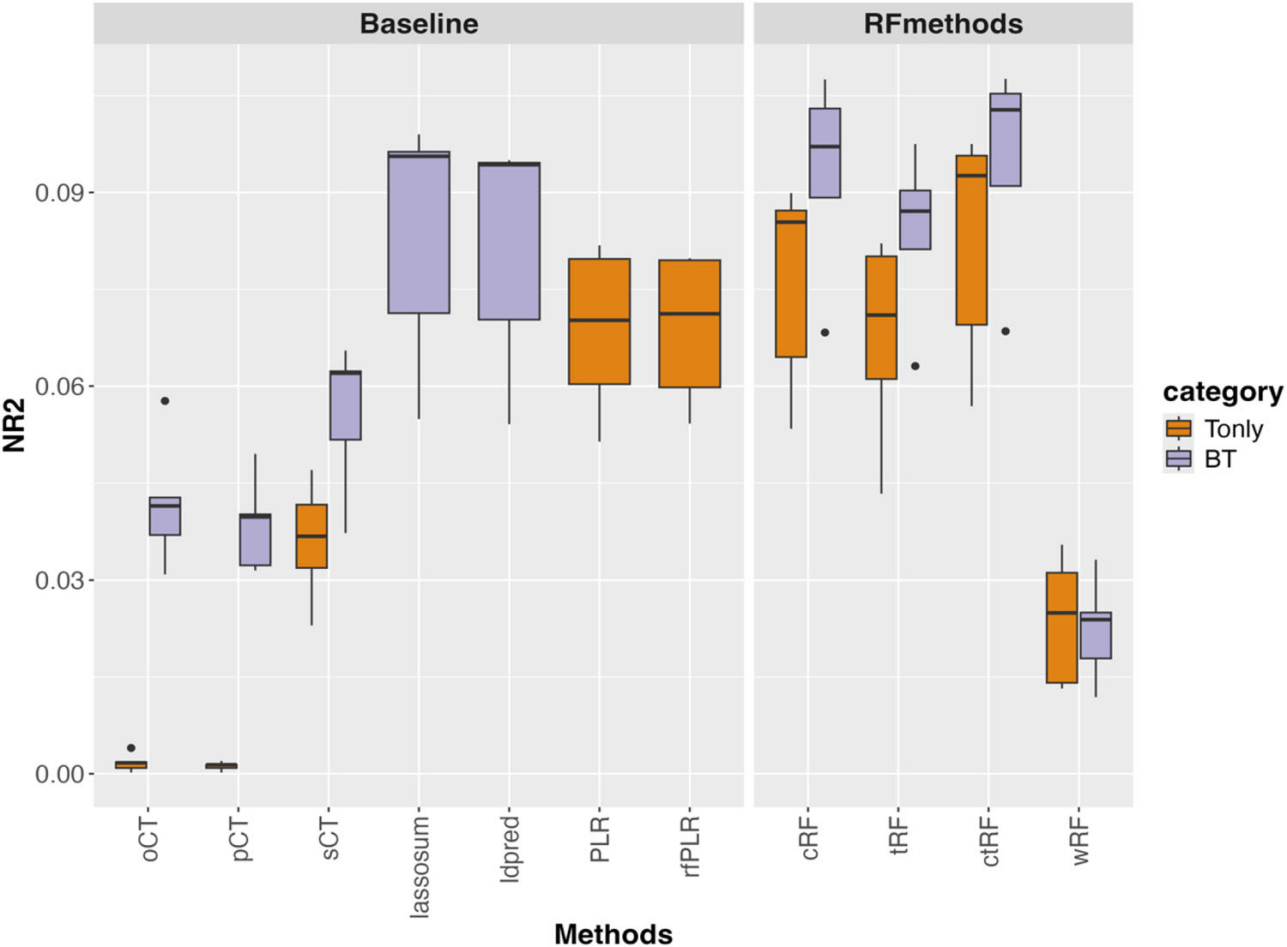
GRS analysis for overweight/obesity, using Taiwan Biobank (TWB) samples (individuals of East Asian descent) as target data and UK Biobank summary statistics (individuals of European descent) as base data. The figure shows boxplots of the Nagelkerke *R* ^2^ (NR2) values for different GRS methods, obtained from 5‐fold cross‐validation.

Figure 7 shows the NR2 of different GRS methods in the overweight/obesity GRS analysis, using TWB samples as target data and UK Biobank samples as base data. The key results can be summarized as follows. (1) In the Tonly analysis, ctRF (along with cRF and tRF) shows slightly higher or similar NR2 values compared to PLR and rfLR (additive models), followed by wRF and sCT, and finally oCT and pCT. These relative NR2s are consistent with the findings from Simulation Scenario 3, suggesting a possible combination of additive and interactive polygenic effects. (2) Compared to Tonly analysis, incorporating base information can further increase NR2 for all methods, suggesting the usefulness of base information even though the base data (European descent) and target data (East Asian descent) are from different ethnic backgrounds. (3) As shown in the AD analysis, methods that incorporate base information and use a joint model framework (e.g., LDpred and lassosum) outperform their Tonly counterparts (e.g., PLR and rfLR). Nevertheless, the BT‐based ctRF still shows slightly higher NR2 than both LDpred and lassosum.

**Figure 8:**
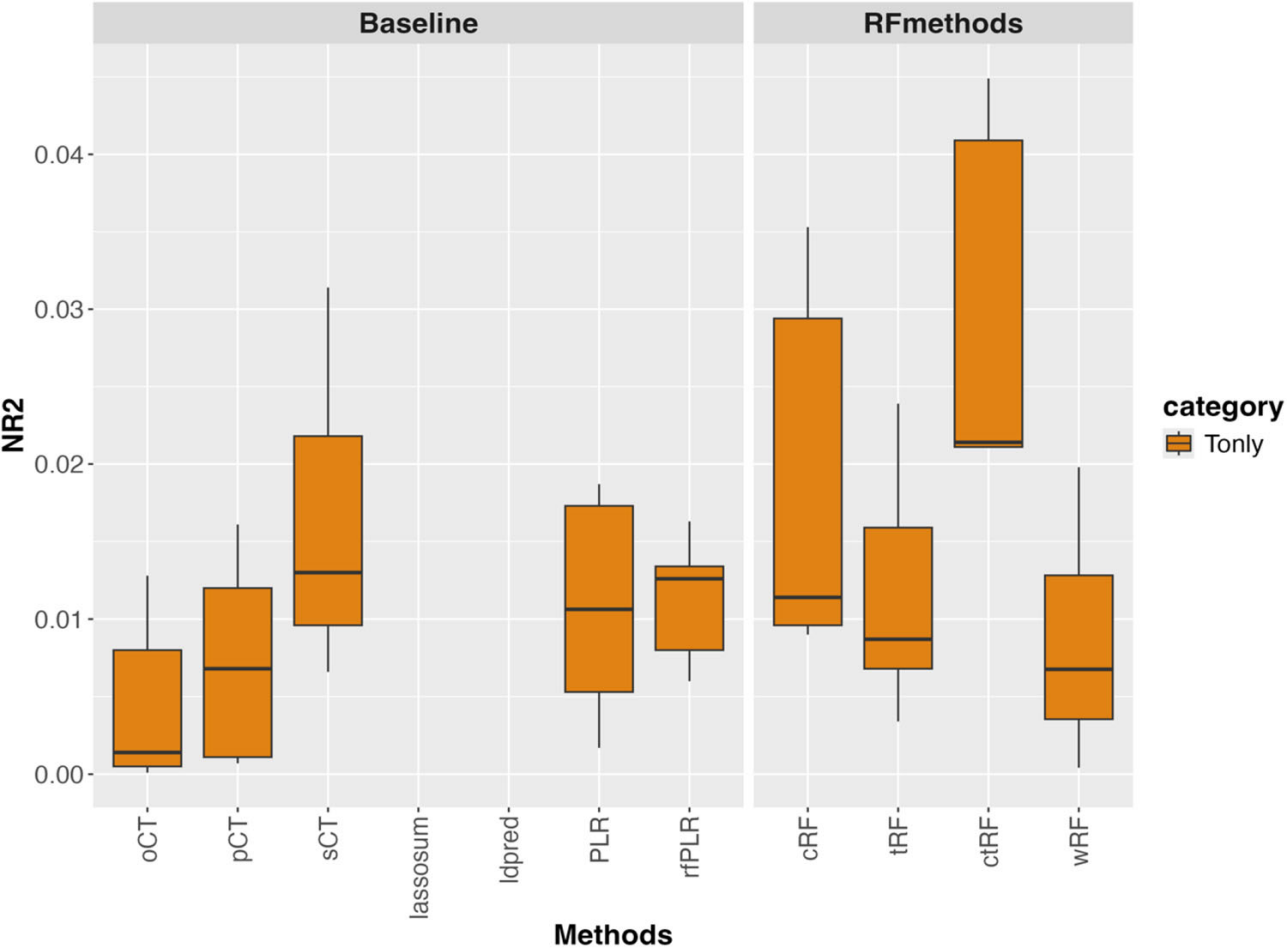
GRS analysis for atopy, using LIGHTS samples (individuals of East Asian descent) as target data for target‐only analysis. The figure shows boxplots of the Nagelkerke *R* ^2^ (NR2) values for different GRS methods, obtained from 5‐fold cross‐validation.

Figure 8 shows the NR2 of different GRS methods in the atopy GRS analysis, using only target data from LIGHTS samples. In this Tonly analysis, ctRF has the highest NR2, followed by cRF as well as additive‐model‐based PLR, rfLR, and sCT, and finally closely followed by tRF, wRF, pCT, and oCT. The relative NR2s are consistent with Simulation Scenario 2, suggesting the possible presence of interactive polygenic effects.

## 4 Discussion

In this paper, we examine the application of RF as a model‐free machine‐learning method for constructing GRS. We use the disease probability estimated from RF models as the GRS for an individual and explore various strategies to implement RF GRS, including ctRF, which optimizes LD clumping cutoff and *p*‐value threshold within RF, and wRF, which up‐/downweights the chance of a SNP being included in a tree node based on its association strength. Our findings show that ctRF performs the best among all RF‐based GRS methods. Similar to classic GRS methods, incorporating informative base data into the construction of RF‐GRS enhances predictive accuracy. In contrast to ctRF, wRF shows limited improvement over baseline methods. Furthermore, incorporating base information often leads to worse performance compared to Tonly analysis, even when the base data are highly informative about target causal variants (e.g., BT100 under the additive genetic mechanism of Simulation Scenario 1).

One possible reason for the underperformance of wRF could be that imposing SNP inclusion weights compromises the randomness and diversity of the trees in the forest. In a standard RF, each tree is built using different subsets of data and features, which reduces correlation among the trees and ensures that the ensemble model benefits from averaging uncorrelated predictions. In wRF, however, SNPs with stronger associations are more likely to be selected in a tree, which may inadvertently reduce tree diversity. Consequently, individual trees in the forest may become correlated, undermining the advantage of ensemble learning. The resulting model may overemphasize the most strongly associated SNPs while overlooking important weaker signals and interactions.

The relative gain of ctRF over cRF and tRF suggests the need of fine‐tuning the LD clumping cutoffs and *p*‐value threshold, especially for LD clumping, which helps to mitigate the limitation of RF when dealing with correlated variables (Gregorutti et al. 2017). We also observe that ctRF incorporating base data can improve upon ctRF using target data only in some scenarios but not always, depending on the similarities between target causal variants and base causal variants and on the underlying effect mechanisms. Therefore, we implement ctRF to include both versions, that is, ctRF‐Tonly (which only uses target data) and ctRF‐BT (which incorporates base data), and users can select the best one and gain robustness against the unknown level of the informativeness of base data according to the underlying genetic effects of target population. The R package “*RF‐GRS*” for performing ctRF is available on GitHub (https://github.com/vvenkat6/RF-GRS).

While our results demonstrate the potential of RF in constructing GRS, several limitations warrant further investigation. First, while ctRF outperforms baseline additive methods when traits are influenced by complex interactive genetic effects, ctRF has lower predictive NR2 compared to the best‐performed methods when traits are influenced by linear additive genetic effects. As expected, the relative performance of different GRS methods varies depending on the genetic architecture and the availability of informative base data. For traits with an additive polygenic effect (e.g., Simulation Scenario 1 and AD analysis), additive GRS models coupling with joint modeling of all SNPs (e.g., PLR, rfLR, LDpred, and lassosum) perform the best, and methods incorporating informative base data (e.g., LDpred and lassosum with BT100 or BT70) show improved predictive accuracy compared to target‐only methods (e.g., PLR and rfLR). On the other hand, when the genetic architecture deviates away from additive effects—such as in cases of interactive effects (e.g., Simulation Scenario 2 and overweight/obesity analysis) or mixed main and interactive effects (e.g., Simulation Scenario 3 and atopy analysis). ctRF shows promise in capturing these complex genetic effects and yields comparable or better predictive accuracy. In practice, it may be beneficial to consider models with complementary genetic architecture assumptions during training and select the optimal model; the final model may also provide insights into the underlying genetic mechanisms.

While RF do provide variable importance measures, the RF framework we employ here—following Malley et al. (2012) probability estimation approach—is explicitly designed for accurate risk prediction rather than for identifying causal SNPs. This approach focuses on producing well‐calibrated disease risk probabilities for individuals, not on selecting specific SNPs that drive disease. Moreover, in high‐dimensional genetic data with strong LD and correlated features, RF models tend to select representative SNPs within LD blocks, making variable importance scores difficult to interpret biologically. These scores can be unstable and may not reliably identify the true causal variants or interactions. Systematically evaluating and refining methods to improve interpretability and recover simulated causal SNPs and interactions remains an important direction for future work that would complement the prediction‐focused analyses presented here.

Additionally, it would be beneficial to explore more sophisticated strategies for SNP weighting in RF beyond the simple association‐based weighting in wRF. An ideal weighting scheme would take advantage of prior information while not negatively impacting tree diversity to enhance RF performance, especially when SNP interactions are crucial.

Finally, our study primarily focused on binary outcomes. For continuous traits, RF can be applied by using the mean or median values in terminal nodes, instead of class probabilities, to generate GRS estimates. While the extension is straightforward, the computational burden can be heavier than for binary traits due to the complexity of evaluating numerous potential splits along continuous variables and the need to average predictions across multiple trees. The overall computational cost increases substantially with the necessity of tuning multiple hyperparameters (e.g., LD clumping cutoffs, *p*‐value thresholds, mtry, and ntree). Future research focusing on optimizing the computational efficiency of RF algorithms for continuous traits, possibly through parallel computing or cloud‐based solutions, would greatly enhance the accessibility of RF‐GRS.

## Supporting information


**Supplementary S1:** Quality Control (QC) protocol of GWAS real data. **Supplementary Table 1:** Summary of all GRS methods considered, including proposed methods and baseline methods. **Supplementary Figure 1:** Boxplots of the AUC values for different GRS methods, based on 100 simulation replications under Simulation Scenario 1, where causal SNPs exhibit additive main effects on the outcome. The target sample size *n*_target_ = 1000. **Supplementary Figure 2:** Boxplots of the AUC values for different GRS methods, based on 100 simulation replications under Simulation Scenario 2, where causal SNPs exhibit two‐way SNP‐SNP interaction effects on the outcome. The target sample size *n*_target_ = 1000. **Supplementary Figure 3:** Boxplots of the AUC values for different GRS methods, based on 100 simulation replications under Simulation Scenario 3, where causal SNPs exhibit a combination of main and interactive effects on the outcome. The target sample size *n*_target_ = 1000. **Supplementary Figure 4:** Boxplots of the AUC values for different GRS methods, based on 100 simulation replications under Simulation Scenario 4, where causal SNPs exhibit three‐way SNP‐SNP interaction effects on the outcomes. The target sample size *n*_target_ = 1000. **Supplementary Figure 5:** Boxplots of the NR2 values (top two rows) and AUC values (bottom two rows) for different GRS methods, based on 100 simulation replications under Simulation Scenario 5a, where causal SNPs have quadratic effects on the outcome and most causal alleles are of low frequency. The target sample size *n*_target_ = 1000. **Supplementary Figure 6:** Boxplots of the NR2 values (top two rows) and AUC values (bottom two rows) for different GRS methods, based on 100 simulation replications under Simulation Scenario 5b, where causal SNPs have quadratic effects on the outcomes and causal allele frequencies > 0.3. The target sample size *n*_target_ = 1000. **Supplementary Figure 7:** Boxplots of the NR2 values (top two rows) and AUC values (bottom two rows) for different GRS methods, based on 100 simulation replications under Simulation Scenario 1, where causal SNPs exhibit additive main effects on the outcome. The target sample size *n*_target_ = 2000. **Supplementary Figure 8:** Boxplots of the NR2 values (top two rows) and AUC values (bottom two rows) for different GRS methods, based on 100 simulation replications under Simulation Scenario 2, where causal SNPs exhibit two‐way SNP‐SNP interaction effects on the outcome. The target sample size *n*_target_ = 2000. **Supplementary Figure 9:** Boxplots of the NR2 values (top two rows) and AUC values (bottom two rows) for different GRS methods, based on 100 simulation replications under Simulation Scenario 3, where causal SNPs exhibit a combination of main and interactive effects on the outcome. The target sample size *n*_target_ = 2000. **Supplementary Figure 10:** Boxplots of the NR2 values (top two rows) and AUC values (bottom two rows) for different GRS methods, based on 100 simulation replications under Simulation Scenario 4, where causal SNPs exhibit three‐way SNP‐SNP interaction effects on the outcomes. The target sample size *n*_target_ = 2000.
