# Supplementary material for: Exploring Random Forest in Genetic Risk Score Construction
Source: Genet Epidemiol. 2025 Oct 25;49(8):e70022. doi: 10.1002/gepi.70022 (PMC12553327; doi:10.1002/gepi.70022)

Supplementary Information
of
Exploring Random Forest in Genetic Risk Score Construction

Venkat et al. (2025)

Supplementary S.1. Quality Control (QC) protocol of GWAS real data

We obtain genotyped target data from the LIGHTS, Taiwan Biobank (TWB), and Alzheimer's Disease Sequencing Project (ADSP) studies. From the raw genotype data, we remove extremely rare SNPs with minor allele frequency (MAF) less than 0.01 and filter out SNPs that deviate from Hardy–Weinberg Equilibrium (HWE), defined as having a Fisher's exact test p-value below 10^{-6}.

For the phenotype of BMI, we use GWAS summary statistics from the UK Biobank as the base dataset. For Alzheimer's disease, we use summary statistics from the International Genomics of Alzheimer's Project (IGAP). For atopy, no external base data are used; the analysis is conducted using the target data alone.

From each base dataset, we apply the same MAF and HWE filters, then match SNPs between the base and target datasets using genomic positions based on the hg38 reference genome. We retain only overlapping SNPs and apply strand flipping and allele reversal as needed to ensure consistency

Supplementary Table 1. Summary of all GRS methods considered, including proposed methods and baseline methods.

**GRS Methods**	**Description**	**Applicability**	
**Proposed Methods**		**Base Data + Target Data**	**Only Target Data**

**ctRF**	· Incorporates LD clumping and p-value thresholding into RF. Final GRS is the disease probability from the RF model with max Nagelkerke R^2 in the training set.<br>· Hyperparameters (p - number of SNPs):<br>– mtry: {sqrt(p)/2, sqrt(p), sqrt(p) * 2, 0.1 * p, 0.5 * p}<br>– ntree: Simulation- {500,1000}; Real Data- 5000<br>– LD R^2 cutoff: Simulation- {0.01, 0.05, 0.1, 0.2, 0.5, 0.8, 0.95}; Real Data- {0.01, 0.05, 0.1, 0.2, 0.5,0.8}<br>– p-value threshold: A sequence of 5 thresholds between the smallest and largest p values, equally spaced on a log-log scale.	✓	✓
**tRF**	Same as ctRF but only considers various p-value thresholding	✓	✓
**cRF**	Same as ctRF but only considers various clumping cutoffs	✓	✓
**wRF**	· Use association strength to up-/down-weigh the chance of a SNP to be included into RF. Final GRS is the disease probability from the RF model with max Nagelkerke R^2<br>· Hyperparameters:<br>– mtry: same as ctRF<br>– ntree: same as ctRF	✓	✓
**Baseline Methods**		**Base Data + Target Data**	**Only Target Data**
**oCT**<br>(Original Clumping and Thresholding)<br>(Choi et al., 2019; Euesden et al., 2015; Purcell et al., 2007)	Selects SNPs that yield max Nagelkerke R^2 in the training set based on p-value thresholds and LD clumping. GRS is calculated as a weighted sum of selected SNPs, weighted by their effect sizes.	✓	✓
**sCT**<br>(Stacked C+T)<br>(Privé, et al., 2019)	Aggregates all GRS for individuals in the training set and fits weights for each C+T score using penalized logistic regression.	✓	✓

**pCT** (PCA-GRS) (Coombes et al., 2020)	Computes GRS under various parameter settings, performs PCA on the resulting set of GRSs, and uses the first principal component as GRS.	✓	✓
**LDpred** (Vilhjálmsson et al., 2015)	A Bayesian method that accounts for SNP LD to estimate SNP effect sizes using summary statistics from base data.	✓	
**lassosum** (Mak et al., 2017)	Utilizes LASSO on summary statistics from base data to promote sparsity in regression coefficients and select SNPs. The non-zero coefficients obtained are then used as weights to compute GRS.	✓	
**PLR** (penalized logistic regression) (Privé et al., 2019)	Logistic regression with elastic net penalty.		✓
**rfLR** (RF screening + Logistic Regression) (Chuang et al., 2017)	Uses RF to identify candidate SNPs for GRS calculation based on the variable importance measures and then uses stepwise logistic regression to further select SNPs and estimate their effect sizes to compute GRS.		✓
**oRF** (Original Random Forest with all SNPs)(Malley et al., 2012)	Trains a Random Forest using all available SNPs without any prior LD clumping or p-value thresholding. Final GRS is the disease probability from the RF model with max Nagelkerke R^2 in the training set.<br>Hyperparameters:<br>− mtry: same as ctRF<br>− ntree: same as ctRF	✓	✓

Supplementary Figure 1: Boxplots of the AUC values for different GRS methods, based on 100 simulation replications under Simulation Scenario 1, where causal SNPs exhibit additive main effects on the outcome. "Tonly" indicates analysis using only target data; "BTq" indicates analysis using both base and target data, where q=100, 70, 50, and 30, denoting the number of causal SNPs shared in common between the base causal SNPs and target causal SNPs out of the 100 base causal SNPs. The target sample size n_{target}=1000.

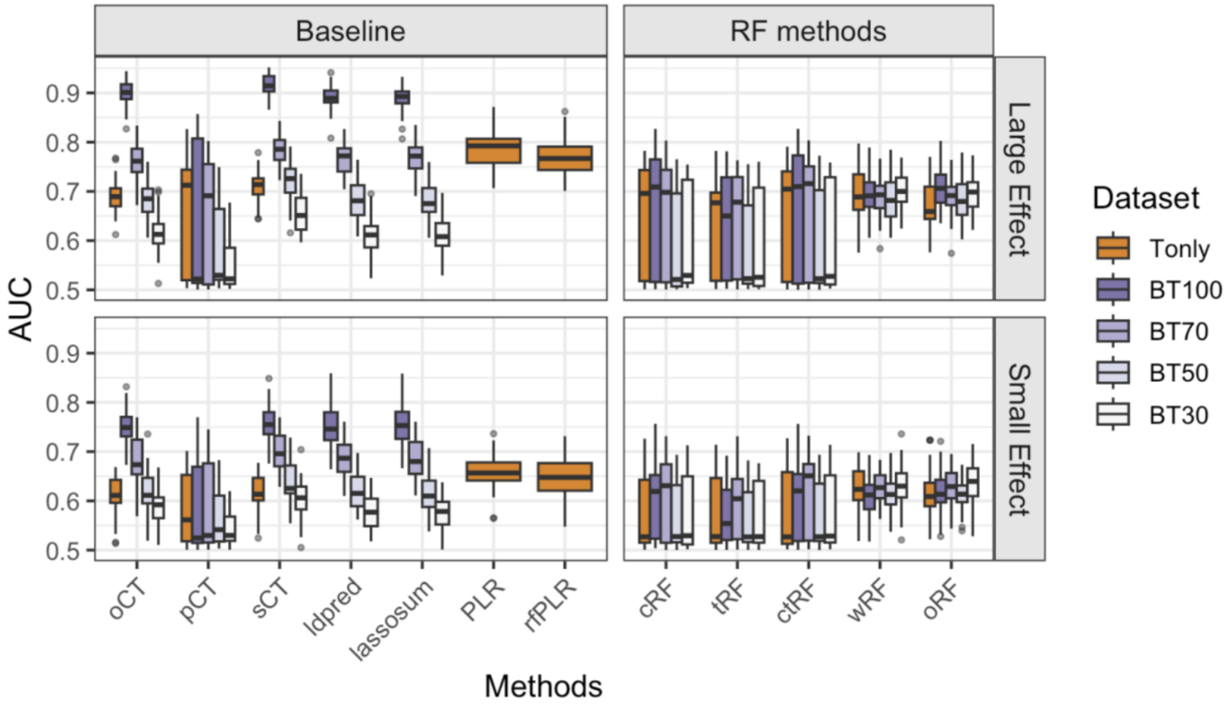

Supplementary Figure 2: Boxplots of the AUC values for different GRS methods, based on 100 simulation replications under Simulation Scenario 2, where causal SNPs exhibit two-way SNP-SNP interaction effects on the outcome. “Tonly” indicates analysis using only target data; “BTq” indicates analysis using both base and target data, where q=100, 70, 50, and 30, denoting the number of causal SNPs shared in common between the base causal SNPs and target causal SNPs out of the 100 base causal SNPs. The target sample size n_{target}=1000.

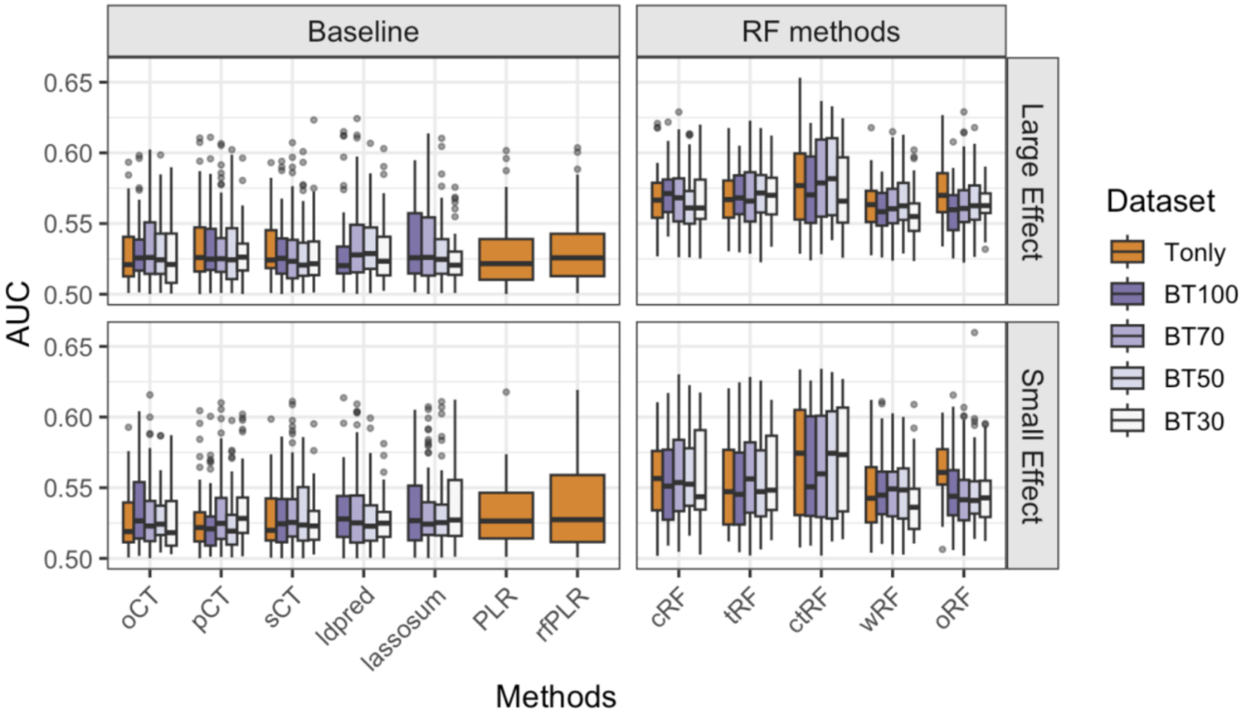

Supplementary Figure 3: Boxplots of the AUC values for different GRS methods, based on 100 simulation replications under Simulation Scenario 3, where causal SNPs exhibit a combination of main and interactive effects on the outcome. "Tonly" indicates analysis using only target data; "BTq" indicates analysis using both base and target data, where q=100, 70, 50, and 30, denoting the number of causal SNPs shared in common between the base causal SNPs and target causal SNPs out of the 100 base causal SNPs. The target sample size n_{target}=1000.

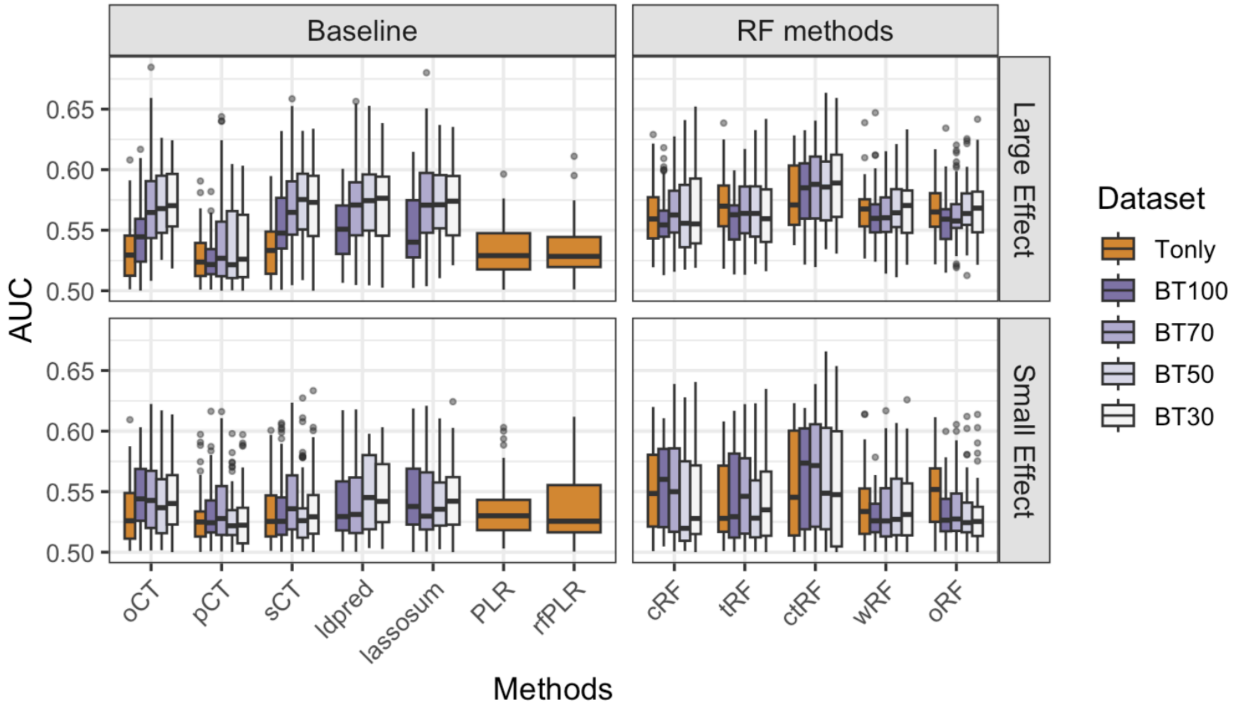

Supplementary Figure 4: Boxplots of the AUC values for different GRS methods, based on 100 simulation replications under Simulation Scenario 4, where causal SNPs exhibit three-way SNP-SNP interaction effects on the outcomes. “Tonly” indicates analysis using only target data; “BTq” indicates analysis using both base and target data, where q=100, 70, 50, and 30, denoting the number of causal SNPs shared in common between the base causal SNPs and target causal SNPs out of the 100 base causal SNPs. The target sample size n_{target}=1000.

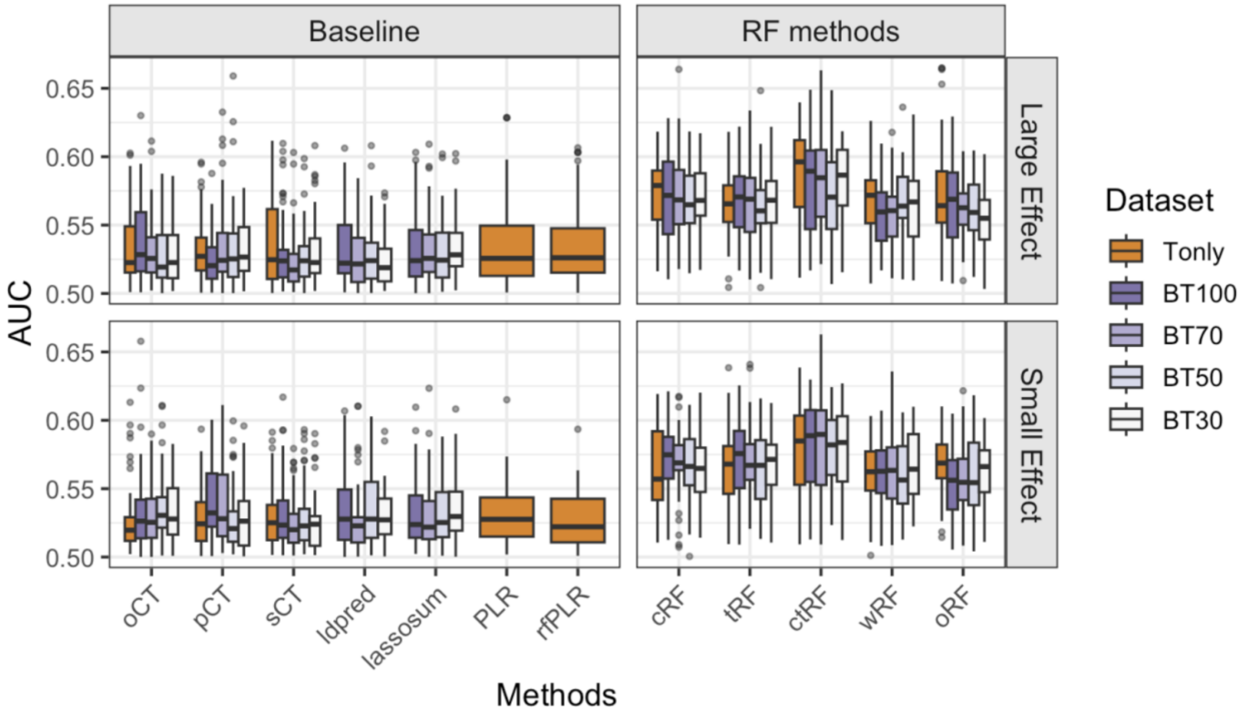

Supplementary Figure 5. Boxplots of the NR2 values (top two rows) and AUC values (bottom two rows) for different GRS methods, based on 100 simulation replications under <u>Simulation Scenario 5a, where causal SNPs have quadratic effects on the outcome and most causal alleles are of low frequency</u>. "Tonly" indicates analysis using only target data; "BTq" indicates analysis using both base and target data, where q=100, 70, 50, and 30, denote the number of causal SNPs shared in common between the base causal SNPs and target causal SNPs out of the 100 base causal SNPs. The target sample size n_{target}=1000.

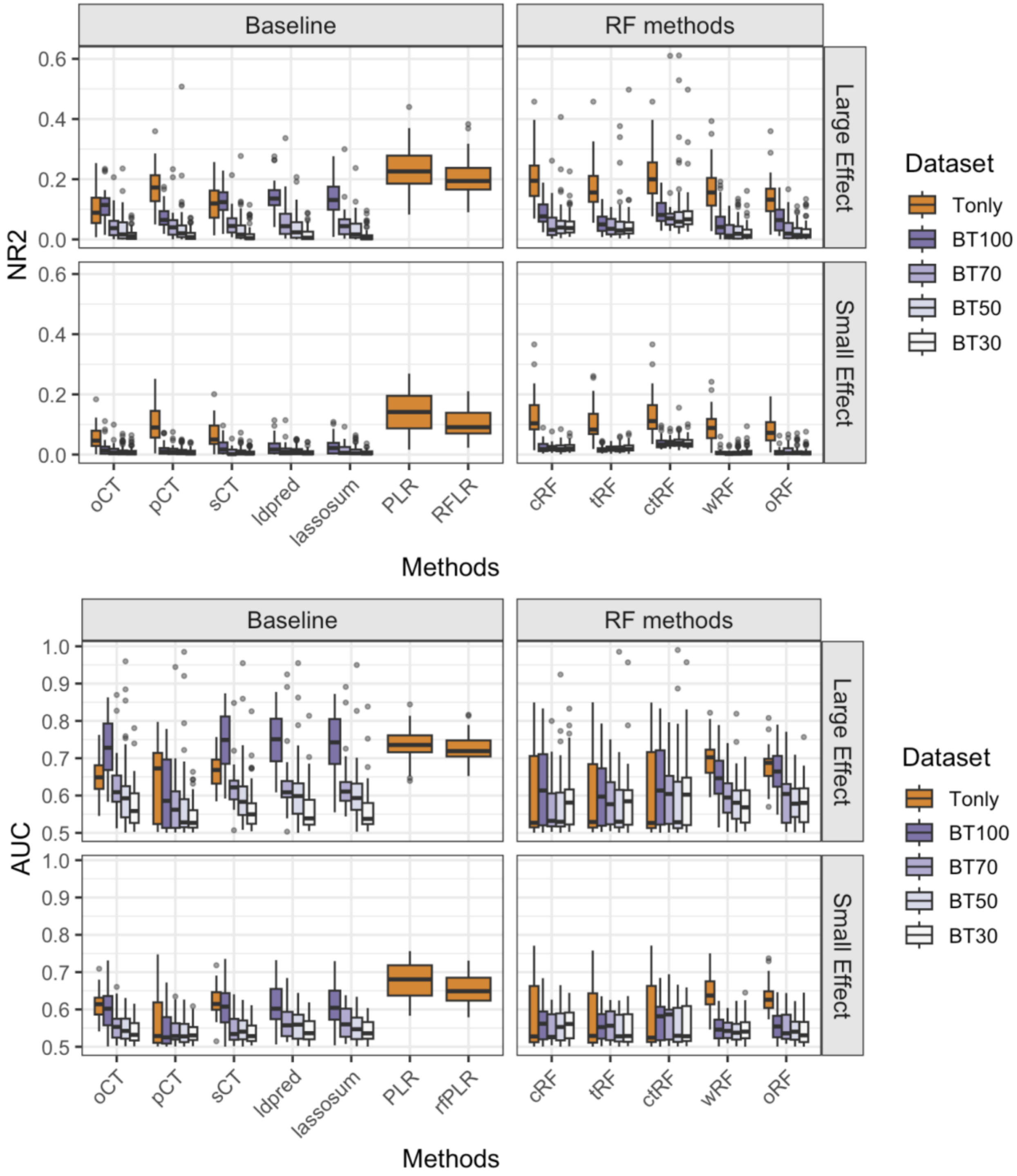

Supplementary Figure 6.: Boxplots of the NR2 values (top two rows) and AUC values (bottom two rows) for different GRS methods, based on 100 simulation replications under Simulation Scenario 5b, where causal SNPs have quadratic effects on the outcomes and causal allele frequencies >0.3. "Tonly" indicates analysis using only target data; "BTq" indicates analysis using both base and target data, where q=100, 70, 50, and 30, denote the number of causal SNPs shared in common between the base causal SNPs and target causal SNPs out of the 100 base causal SNPs. The target sample size n_{target}=1000.

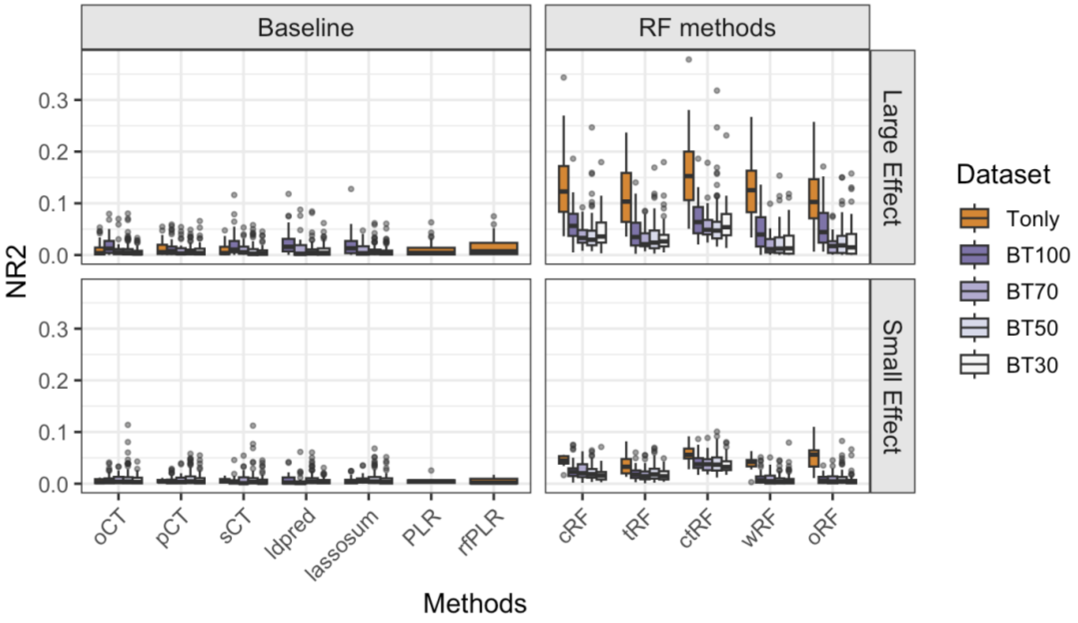

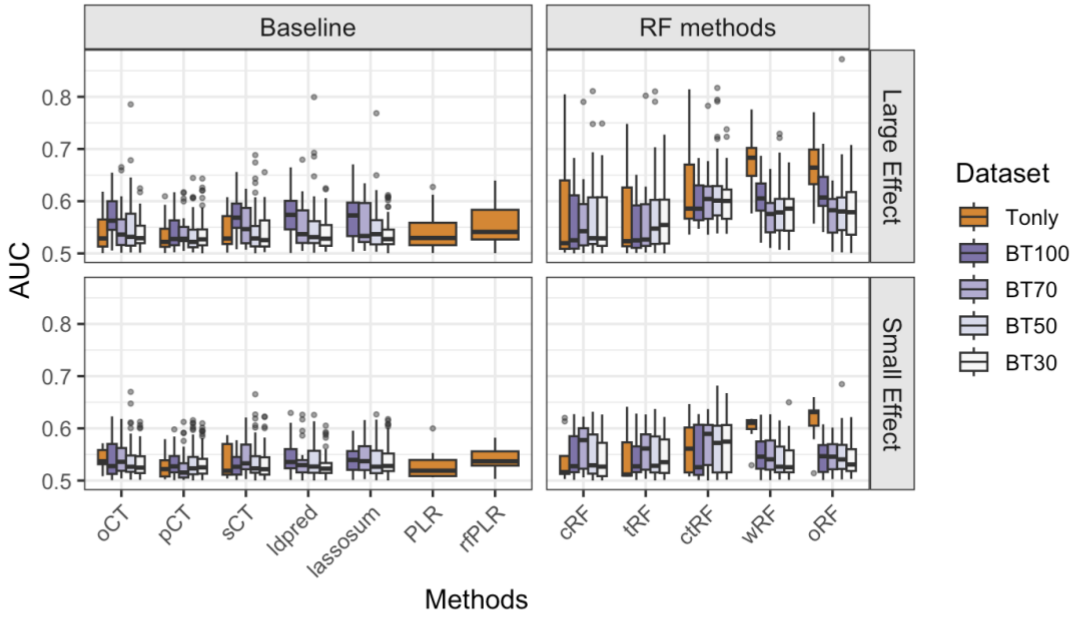

Supplementary Figure 7. Boxplots of the NR2 values (top two rows) and AUC values (bottom two rows) for different GRS methods, based on 100 simulation replications under Simulation Scenario 1, where causal SNPs exhibit additive main effects on the outcome. “Tonly” indicates analysis using only target data. “Tonly” indicates analysis using only target data; “BTq” indicates analysis using both base and target data, where q=100, 70, 50, and 30, denote the number of causal SNPs shared in common between the base causal SNPs and target causal SNPs out of the 100 base causal SNPs. The target sample size n_{target}=2000.

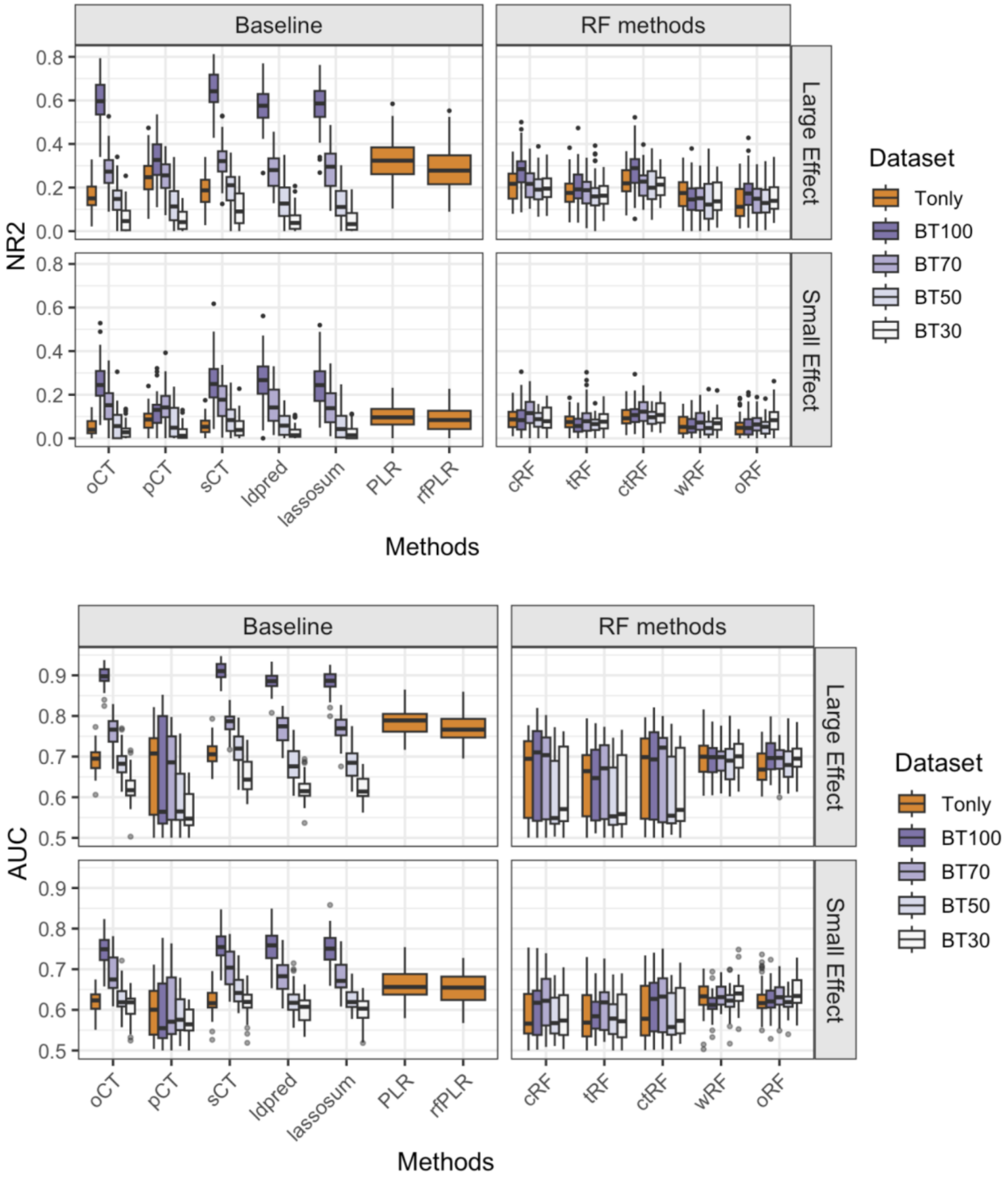

Supplementary Figure 8. Boxplots of the NR2 values (top two rows) and AUC values (bottom two rows) for different GRS methods, based on 100 simulation replications under Simulation Scenario 2, where causal SNPs exhibit two-way SNP-SNP interaction effects on the outcome. "Tonly" indicates analysis using only target data. "Tonly" indicates analysis using only target data; "BTq" indicates analysis using both base and target data, where q=100, 70, 50, and 30, denote the number of causal SNPs shared in common between the base causal SNPs and target causal SNPs out of the 100 base causal SNPs. The target sample size n_{target}=2000.

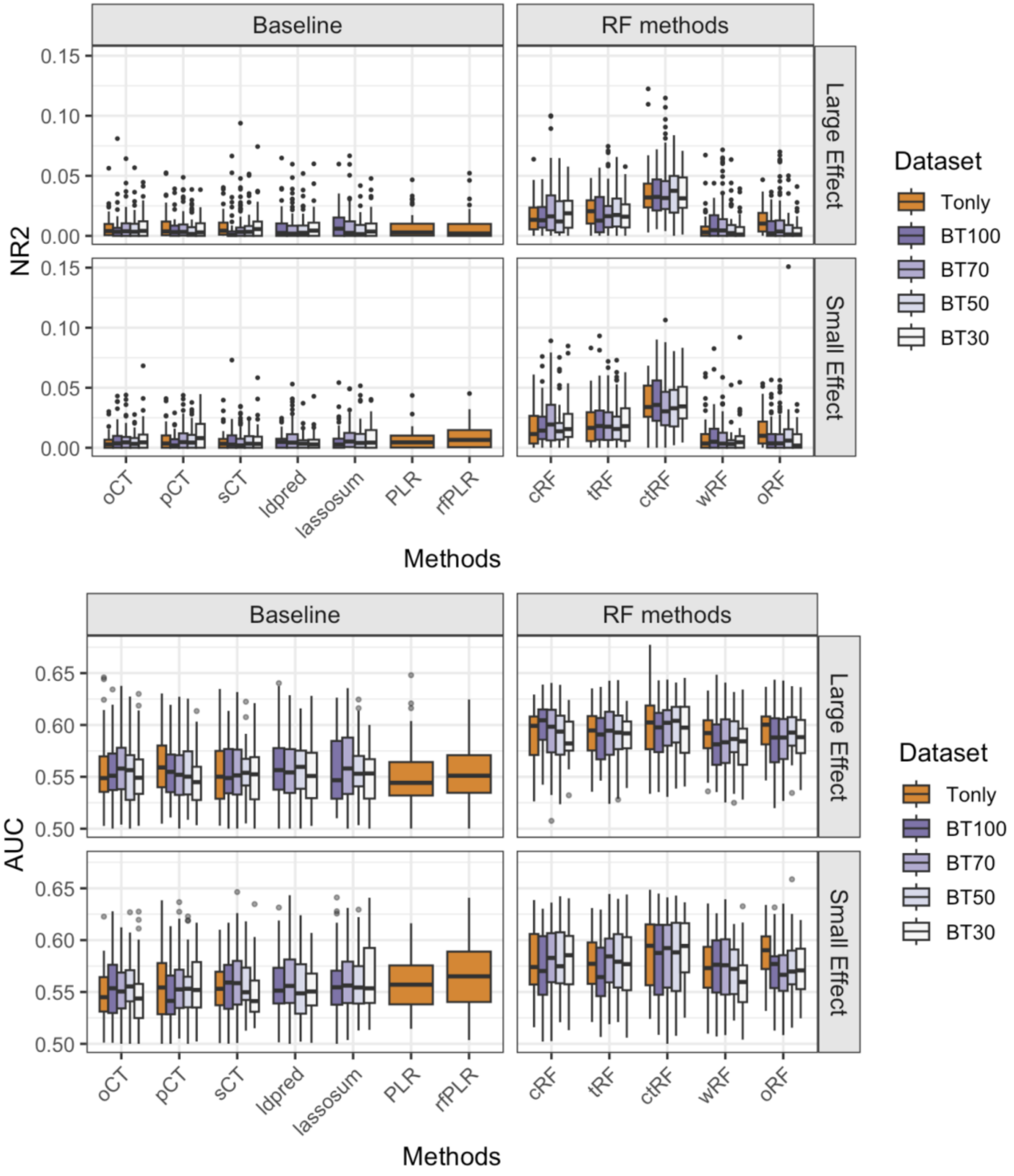

Supplementary Figure 9. Boxplots of the NR2 values (top two rows) and AUC values (bottom two rows) for different GRS methods, based on 100 simulation replications under Simulation Scenario 3, where causal SNPs exhibit a combination of main and interactive effects on the outcome. "Tonly" indicates analysis using only target data. "Tonly" indicates analysis using only target data; "BTq" indicates analysis using both base and target data, where q=100, 70, 50, and 30, denote the number of causal SNPs shared in common between the base causal SNPs and target causal SNPs out of the 100 base causal SNPs. The target sample size n_{target}=2000.

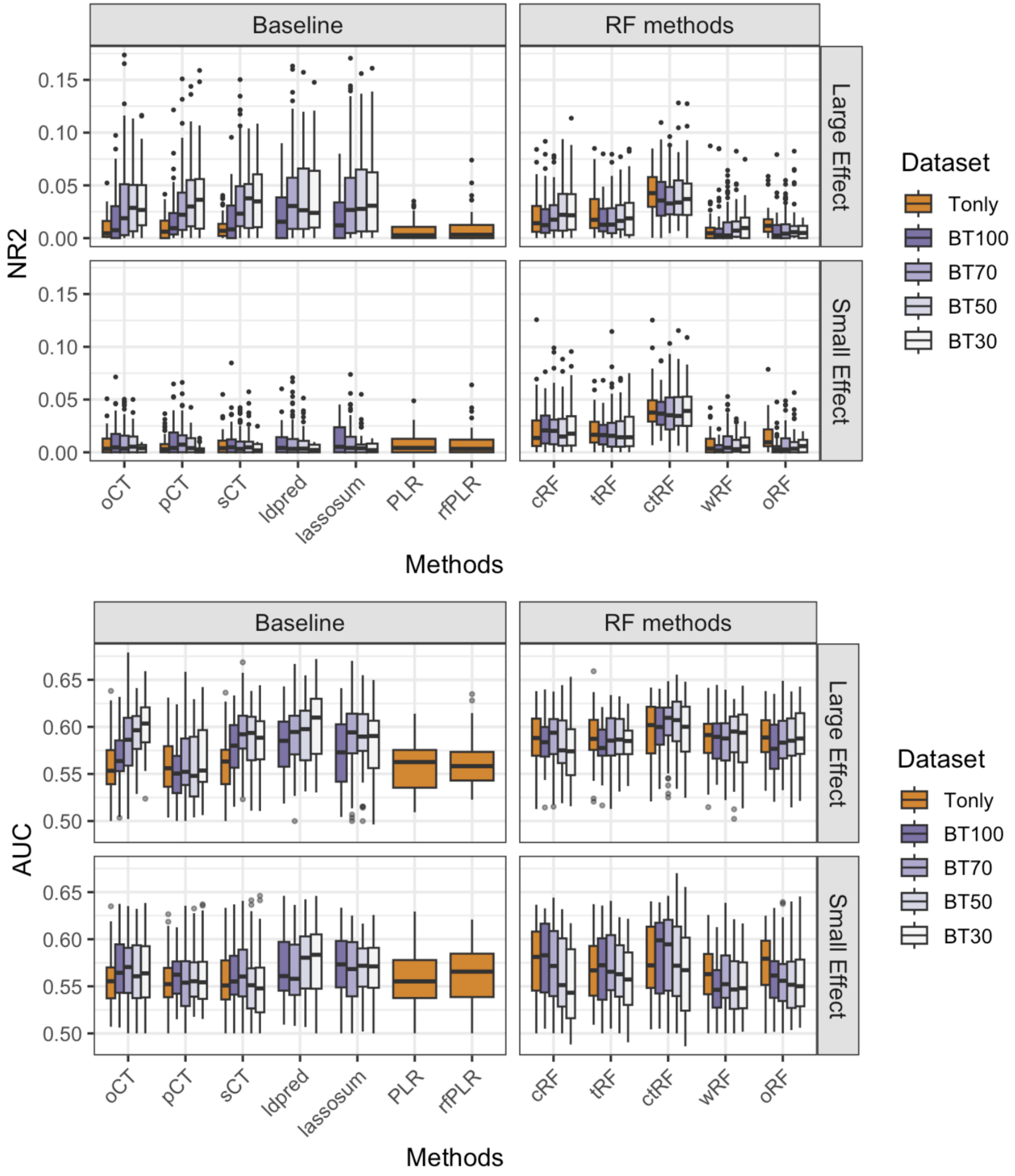

Supplementary Figure 10. Boxplots of the NR2 values (top two rows) and AUC values (bottom two rows) for different GRS methods, based on 100 simulation replications under Simulation Scenario 4, where causal SNPs exhibit three-way SNP-SNP interaction effects on the outcomes. "Tonly" indicates analysis using only target data. "Tonly" indicates analysis using only target data; "BTq" indicates analysis using both base and target data, where q=100, 70, 50, and 30, denote the number of causal SNPs shared in common between the base causal SNPs and target causal SNPs out of the 100 base causal SNPs. The target sample size n_{target}=2000.

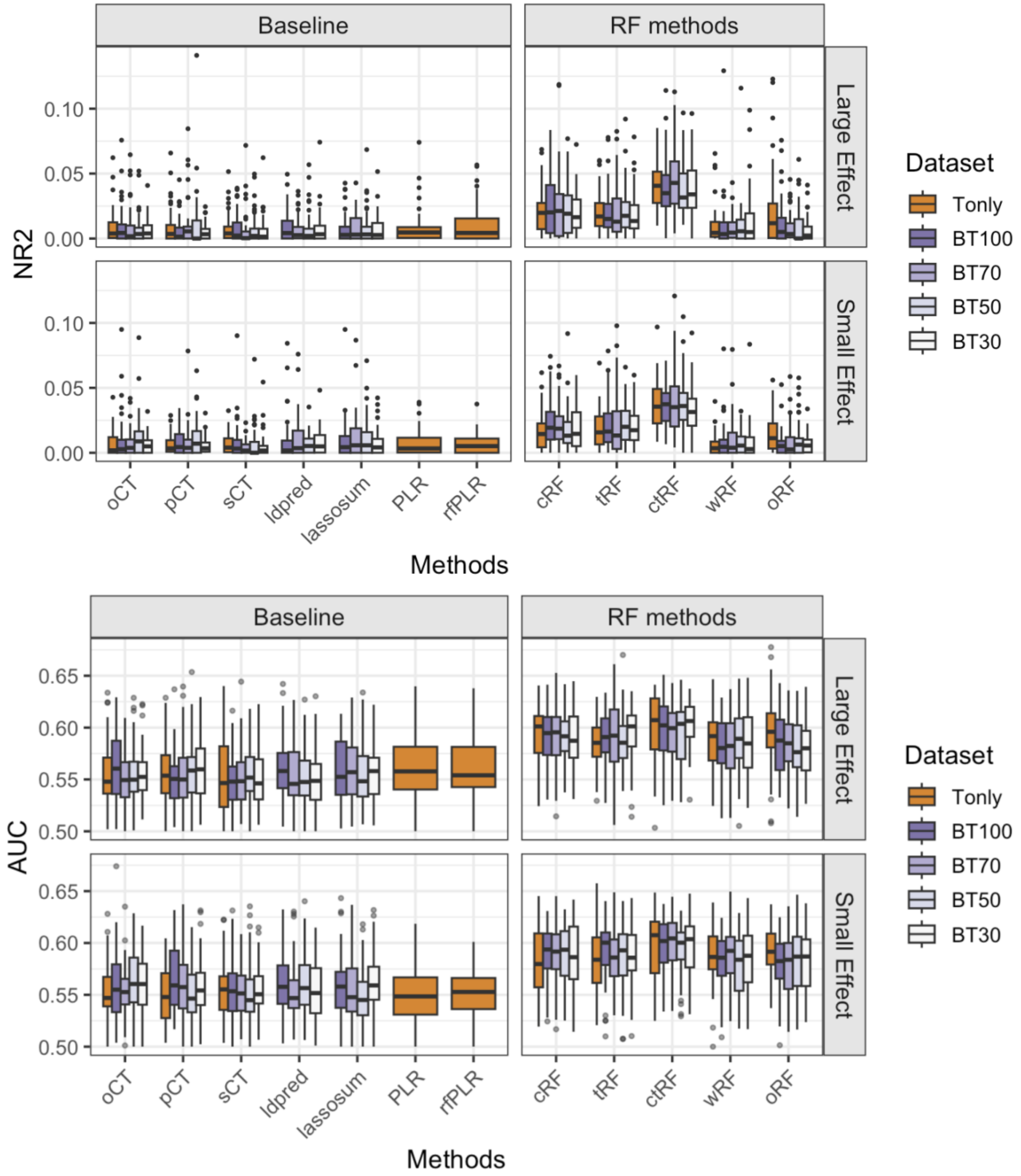

Supplement: Supplementary file 1 — Supplementary S1: Quality Control (QC) protocol of GWAS real data. Supplementary Table 1: Summary of all GRS methods considered, including proposed methods and baseline methods. Supplementary Figure 1: Boxplots of the AUC values for different GRS methods, based on 100 simulation replications under Simulation Scenario 1, where causal SNPs exhibit additive main effects on the outcome. The target sample size n target = 1000. Supplementary Figure 2: Boxplots of the AUC values for different GRS methods, based on 100 simulation replications under Simulation Scenario 2, where causal SNPs exhibit two‐way SNP‐SNP interaction effects on the outcome. The target sample size n target = 1000. Supplementary Figure 3: Boxplots of the AUC values for different GRS methods, based on 100 simulation replications under Simulation Scenario 3, where causal SNPs exhibit a combination of main and interactive effects on the outcome. The target sample size n target = 1000. Supplementary Figure 4: Boxplots of the AUC values for different GRS methods, based on 100 simulation replications under Simulation Scenario 4, where causal SNPs exhibit three‐way SNP‐SNP interaction effects on the outcomes. The target sample size n target = 1000. Supplementary Figure 5: Boxplots of the NR2 values (top two rows) and AUC values (bottom two rows) for different GRS methods, based on 100 simulation replications under Simulation Scenario 5a, where causal SNPs have quadratic effects on the outcome and most causal alleles are of low frequency. The target sample size n target = 1000. Supplementary Figure 6: Boxplots of the NR2 values (top two rows) and AUC values (bottom two rows) for different GRS methods, based on 100 simulation replications under Simulation Scenario 5b, where causal SNPs have quadratic effects on the outcomes and causal allele frequencies > 0.3. The target sample size n target = 1000. Supplementary Figure 7: Boxplots of the NR2 values (top two rows) and AUC values (bottom two rows) for different [file GEPI-49-0-s001.pdf]
